# Co-designing digital nudges to promote gender equality and social inclusion among Arab youth: a mixed-methods study

**DOI:** 10.3389/fpsyg.2026.1763151

**Published:** 2026-09-08

**Authors:** Alaa Ziyud, Khaled AlThelaya, Jens Schneider

**Affiliations:** College of Science and Engineering, Hamad Bin Khalifa University, Doha, Qatar

**Keywords:** Arab youth, behavioral design, co-design, digital nudging, gender equality, mixed-methods, social inclusion

## Abstract

**Background:**

Digital nudges have been widely explored in privacy, health, and sustainability domains; however, little empirical work has co-designed culturally grounded nudges that address Gender Equality and Social Inclusion (GESI), especially in Arab contexts. This gap is particularly significant given the strong influence of cultural norms and socio-economic structures in Arab societies, which shape how GESI are perceived and enacted in digital spaces. This study presents a full co-design process that integrates behavioral insights and participant-led design activities to develop a digital-nudging framework aimed at promoting GESI among Arab youth in Qatar.

**Methods:**

With ethical approval from the Hamad Bin Khalifa University Institutional Review Board (HBKU-IRB-2024-30), a sequential mixed-methods design was employed consisting of three phases: (1) protocol development and pilot testing, (2) data collection through a social-media behavior survey and co-design workshops, and (3) integrated qualitative–quantitative analysis. The Social Media Activity Questionnaire (SMAQ) was used to classify participants' passive and active social-media behaviors (*n* = 32), serving as a preparatory step for grouping participants and informing design activities. Qualitative data were gathered across four co-design workshops.

**Results:**

Survey findings revealed a marked imbalance between passive and active engagement, with passive behaviors (e.g., viewing stories, reading messages) dominating over active content creation. This behavioral insight informed the structuring of co-design groups and the selection of nudge targets. Across the workshops, participants collaboratively generated twelve nudge prototypes emphasizing personalization, messenger effects, and simplification. Participants consistently prioritized transparency, cultural sensitivity, and relatable messaging, highlighting the value of participatory approaches in GESI-oriented digital design.

**Conclusion:**

This study demonstrates a culturally grounded co-design process for developing digital nudges that support GESI. Using behavioral data to guide workshop preparation, the study offers a replicable, user-centered framework for designing responsible and contextually relevant digital interventions for Arab youth.

## 1 Introduction

Promoting Gender Equality and Social Inclusion (GESI) is a core target of the United Nations Sustainable Development Goals (Kelles-Viitanen and Shrestha, 2011). GESI refers to the absence of discrimination in access to opportunities and the full participation of all individuals in society (Stewart et al., 2021). Despite worldwide progress, structural barriers continue to marginalize women and minority groups, particularly within Arab societies where institutional and cultural constraints persist (International WV, 2023; Evidence for Inclusive Development, 2021; Sadiqi, 2017). For example, female labor-force participation in Qatar remains below 30% (International WV, 2023).

Social Networking Sites (SNSs) such as Twitter, Facebook, and Instagram have transformed communication and activism, amplifying initiatives like #HeForShe and #MeToo, that advocate for equality and inclusion (Quan-Haase et al., 2021; Engström, 2019). These platforms can both challenge and reinforce existing social hierarchies, providing opportunities for advocacy yet often result in temporary awareness without lasting behavioral change (Grzyb and Kulczyk, 2023; Reneses and Bosch, 2023). This discrepancy between awareness and action underscores the need for strategies that sustain inclusive digital engagement. While hashtags raise momentary visibility, they seldom translate into sustained behavioral change; nudging offers a mechanism to embed inclusive norms into everyday platform interactions (Özdemir, 2020). Digital nudging, rooted in behavioral economics, subtly guides user decisions without restricting autonomy (Özdemir, 2020; Santiago Walser et al., 2019). In digital spaces, they have influenced behaviors related to privacy (Bergram et al., 2020), smartphone use (Monge Roffarello and De Russis, 2023), and online participation (Santiago Walser et al., 2019). However, most digital-nudge studies focus on privacy, health, or environmental outcomes in Western samples (Bergram et al., 2020; Monge Roffarello and De Russis, 2023; Santiago Walser et al., 2019), leaving a paucity of GESI-focused work in Arab settings. Ethical and cultural considerations—such as autonomy, transparency, and sensitivity to local norms—complicate the transfer of nudge frameworks across cultures (Schmidt and Engelen, 2020; Kuyer and Gordijn, 2023). Existing models, including the Digital Nudging Process Model (DINU) and Human-Centered Design (HCD), provide structure but inadequately capture these sociocultural dimensions (Santiago Walser et al., 2019; Sumner et al., 2023; Abras et al., 2004).

This study addresses this gap by presenting the first empirical co-design of digital nudges that promote GESI among Arab youth. The main goal is to co-design a culturally grounded digital-nudge framework for GESI. Using a participatory design process that combines surveys and co-design workshops, the study develops culturally responsive digital nudges suited to Qatar's social-media context from the perspective of end users. By integrating behavioral design with co-design methodology, the research advances a replicable framework for fostering equitable and inclusive online interactions (Pearson et al., 2020; Asbjørnsen et al., 2022; Trischler et al., 2018).

## 2 Theoretical background

The term “nudge,” coined by Thaler and Sunstein, refers to elements of choice architecture that subtly alter behaviors without restricting options or economic incentives (Thaler and Sunstein, 2008a). In digital contexts, “digital nudging” leverages design, information, and interaction elements to guide user behavior in digital environments without limiting freedom of choice (Bergram et al., 2022). Digital nudging has proven effective across domains such as technology, healthcare, and environmental sustainability. For example, digital nudges have been employed to promote mental health (Grové, 2021), increase vaccination adherence (Parsons et al., 2021), encourage healthier lifestyles (Asbjørnsen et al., 2022), and support sustainable consumption (Purohit et al., 2023). The body of research on digital nudges has expanded significantly, as noted by Bergram et al. (2022), with investigations into their impact on aspects like user privacy behavior (Ioannou et al., 2021) and pro-environmental behavior (Zimmermann et al., 2021). Digital nudges encompass a wide range of strategies that subtly influence behavior. Valta and colleagues' exhaustive examination has delineated ten distinct forms of digital nudges (Valta et al., 2022). These forms exhibit a broad spectrum of approaches, each tailored to distinct mechanisms and objectives. Some endeavors within this domain seek to shape perception and decision-making processes, while others delve into the realm of subconscious influence and motivation. Additionally, certain strategies concentrate on establishing cognitive anchors and facilitating simplified pathways, while others prioritize fostering autonomy and refining decision architecture.

Within the sphere of perception and decision-making, framing emerges as a prominent technique, intricately weaving narratives to mold individuals' interpretations and responses to presented information (Valta et al., 2022). Simultaneously, Status Quo Bias capitalizes on human inertia, steering individuals toward maintaining existing behaviors or choices (Valta et al., 2022). Messenger Effects, on the other hand, harness the persuasive potential of message delivery methods, strategically utilizing trusted messengers and communication channels to enhance message efficacy (Valta et al., 2022). In the domain of subconscious influence and motivation, Priming subtly exposes individuals to stimuli to evoke specific thoughts or behaviors, operating beneath conscious awareness (Valta et al., 2022). Conversely, Loss Aversion emphasizes potential losses to galvanize action, accentuating the consequences of inaction to prompt proactive decision-making (Valta et al., 2022). Hyperbolic Discounting exploits individuals' inclination toward immediate rewards over delayed gratification, influencing choices that prioritize short-term gains (Valta et al., 2022). Understanding these nudge forms is essential to design effective interventions. Research often uses a combination of nudges to optimize behavior change. For example, studies have combined loss aversion, simplification, and default nudges to improve adherence to health recommendations (Parsons et al., 2021). This study integrates digital nudging, DINU, and HCD into a unified co-design framework. While DINU provides a structured approach to nudge development and HCD ensures user-centered alignment, co-design extends this by enabling active user collaboration in shaping interventions. This integration allows co-designed nudges to influence GESI-related behaviors by aligning behavioral mechanisms with users' experiences, enhancing cultural relevance and promoting inclusive online engagement.

### 2.1 Digital nudge

**Figure 1:**
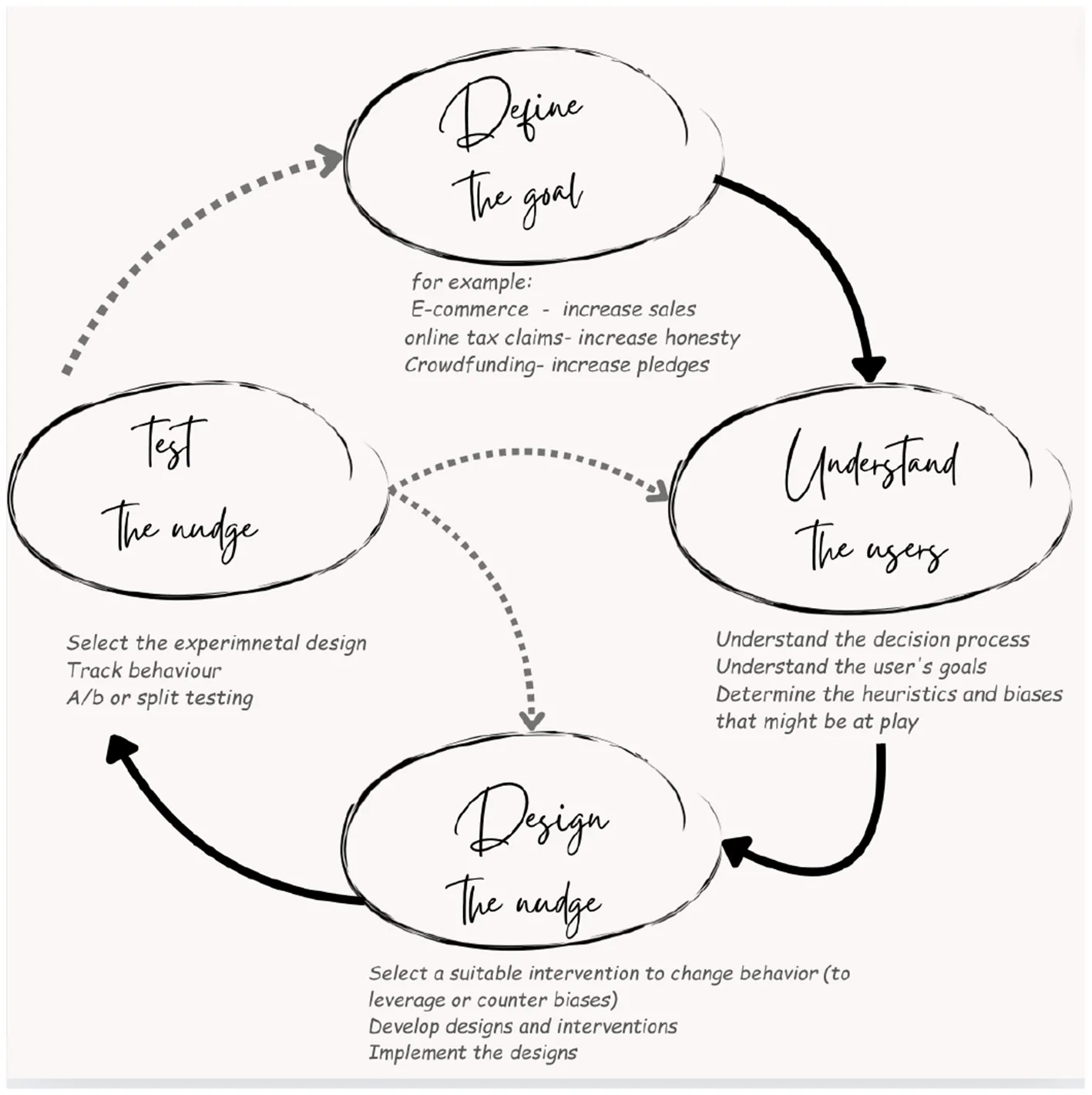
Design process for digital nudges on online platforms, redrawn and adapted from Schneider et al. (2017).

Digital nudges are strategically designed elements embedded in user interfaces to subtly influence users toward making decisions that align with desirable outcomes (Schneider et al., 2017; Ioannou et al., 2021; Zimmermann et al., 2021). Schneider's work, building upon established guidelines for offline nudges, provides a foundation for designing digital nudges. He developed a set of guidelines specifically tailored for digital platforms, illustrated in Figure 1 (Schneider et al., 2017). The process follows a familiar cycle of developing information systems, with stages such as planning, analysis, design, and implementation, and features an iterative nature. The design process begins by defining the objectives and scope of the intervention, identifying the target behaviors and setting guiding goals. This phase leads to the collection and analysis of data to understand user interactions with digital platforms. By studying user behavior, preferences, and challenges, designers can create nudges that are tailored to the target audience. The nudge is then integrated into the platform, ensuring that it functions effectively and influences user behavior (Schneider et al., 2017).

In designing digital nudges, researchers draw on various methodologies, recognizing that different contexts and user needs demand different strategies. Schneider's foundational approach provides the basis for subsequent methods, which can be seen as distinct yet complementary strategies designed to meet specific needs. One such method is the Digital Nudging Process Model (DINU), developed by Santiago Walser et al. (2019). DINU focuses on enhancing decision-making processes on convergence platforms by guiding the design and implementation of digital nudges. The model consists of three key phases: defining goals and reasons, selecting design elements, and implementing and evaluating the nudge (Santiago Walser et al., 2019). By following this structured approach, designers can develop nudges that influence user behavior in predictable ways (Santiago Walser et al., 2019). While DINU serves as a useful tool for guiding digital nudge design across diverse contexts, it does not fully address user preferences and needs (Barev et al., 2021). In contrast, HCD prioritizes user involvement throughout the design process, ensuring that the interventions are closely aligned with user preferences. This approach emphasizes the importance of incorporating small data—digital footprints generated through everyday activities such as online purchases, emails, and commuting—into the design process (Okeke et al., 2018). By leveraging small data, HCD facilitates the creation of personalized nudges that are better suited to individual user needs. This shift represents a significant advancement in behavioral intervention research, as exemplified by Okeke et al. (2018), who developed a mobile intervention that analyzes users' interactions with their devices to reduce excessive screen time. However, while HCD incorporates user preferences into the design, it is not without its drawbacks. For example, biases in user input may lead to incomplete or skewed insights, and the lengthy development cycles associated with HCD can delay intervention implementation (Abras et al., 2004). One promising approach to address these concerns is employing co-design in digital nudge design, which involves actively engaging users in the design process to ensure their perspectives and values are integrated into the development of nudges (Valta et al., 2022). This combined approach expands the focus from the “how to design” of digital nudges to include the “who will design,” emphasizing user agency and collaboration (Sanders and Stappers, 2008). Involving users in the design process enhances the relevance of digital nudges, fosters a sense of ownership, and ultimately leads to more effective and ethically sound interventions (Sanders and Stappers, 2008; Trischler et al., 2018).

### 2.2 Co-design

While participatory design broadly refers to involving users in the design process (Spinuzzi, 2005), co-design represents a more specific approach in which stakeholders—such as designers, users, and other relevant parties—work together to develop and refine digital nudges (Richardson and John, 2021). This process ensures that the result nudges are not only effective in achieving their intended goals but also ethically sound, considering crucial aspects like transparency and user autonomy (Richardson and John, 2021). A core tenet of participatory design is the recognition that those affected by a design should have a voice in its creation (Ehn, 2008). This aligns closely with the principles of human-centered design, which emphasizes user involvement at every stage of the design process (Sanders and Stappers, 2008). Defining participatory design comprehensively can be challenging, but Sanders and Stappers (2008) characterizes it as an approach that invites users—those who will benefit from the design—to actively participate in the process. Expanding on this, Ehn (2008) argues that anyone involved in the design process can be considered a co-designer. Trischler et al. (2018) further highlights the importance of interaction between users and other stakeholders, asserting that participatory design entails collaboration with designers, researchers, and developers. Overall, these perspectives converge on the idea that participatory design actively engages users, addressing the ethical concerns related to influencing individuals without their explicit awareness. The relevance of co-design in mitigating ethical concerns about digital nudging is crucial. Findings from the SMAQ segmentation were triangulated with workshop artifacts, confirming a marked active–passive engagement gap. These profiles anchored the development of the three nudge forms—personalization, messenger effect, and simplification—by aligning prototype intent with empirically observed user-interaction tendencies. By involving users and other stakeholders, co-design fosters transparency and accountability (Sanders and Stappers, 2008). This active engagement ensures that users' preferences, values, and concerns are incorporated into the design process, making nudges more ethically aligned with user needs and aspirations. For these reasons, this study adopts a co-design approach to ensure the development of responsible and context-sensitive digital nudges.

### 2.3 Rationale of the study

In recent years, there has been increasing recognition of the importance of promoting GESI in digital environments, particularly across social media platforms that shape everyday communication and social norms (Guo and Saxton, 2014; Scott and Maryman, 2016; Bowen et al., 2017). While such initiatives have gained traction in Western contexts, GESI issues remain underexplored in the Arab world despite their growing relevance. Reports such as World Vision's GESI Theory of Change and the ECID Framework highlight the persistent structural and cultural barriers to gender and social equity in Arab societies, including limited institutional support and resource constraints (International WV, 2023; Evidence for Inclusive Development, 2021). Moreover, research by Sadiqi (2017) underscores how sociocultural traditions and academic discourse intersect to influence perceptions of women's rights and inclusion in these regions. Given that digital platforms often mirror—and can either reinforce or challenge—existing social hierarchies (Van Dijck, 2020), there is a pressing need to design interventions that encourage equitable participation and inclusive expression online. Recognizing this potential, the present study leverages the power of co-designed digital nudges to promote GESI-oriented behavior among Arab youth. Through a participatory design process that involves surveys and co-design workshops, the study engages young adults in Qatar to collaboratively design digital nudges that foster GESI. In the Arab context, macro-level socio-cultural constraints—such as gender norms, institutional barriers, and societal expectations—shape how individuals engage in digital spaces. These structural inequalities often translate into micro-level behavioral patterns, where users may exhibit more passive engagement, self-censorship, or selective participation when interacting with gender-related content online. For example, concerns around social judgment or cultural sensitivity can discourage active expression, particularly among marginalized groups, while reinforcing cautious or observational behaviors (Raïq et al., 2025; Jwaniat et al., 2025). Understanding this linkage is essential for designing digital nudges that are not only behaviorally effective but also culturally responsive and capable of promoting more inclusive and equitable online interactions.

This research fills a critical gap by presenting the first empirical application of co-design and digital nudging techniques tailored to the Arab cultural context. Ethical approval was obtained from the Institutional Review Board of Hamad Bin Khalifa University (Reference: HBKU-IRB-2024-30), ensuring adherence to institutional and international research standards. This foundation underscores the study's methodological rigor and its contribution to evidence-based approaches for promoting GESI through culturally responsive digital design from the perspective of end users.

## 3 Data collection methods

### 3.1 Framework development and intervention workflow

**Figure 2:**
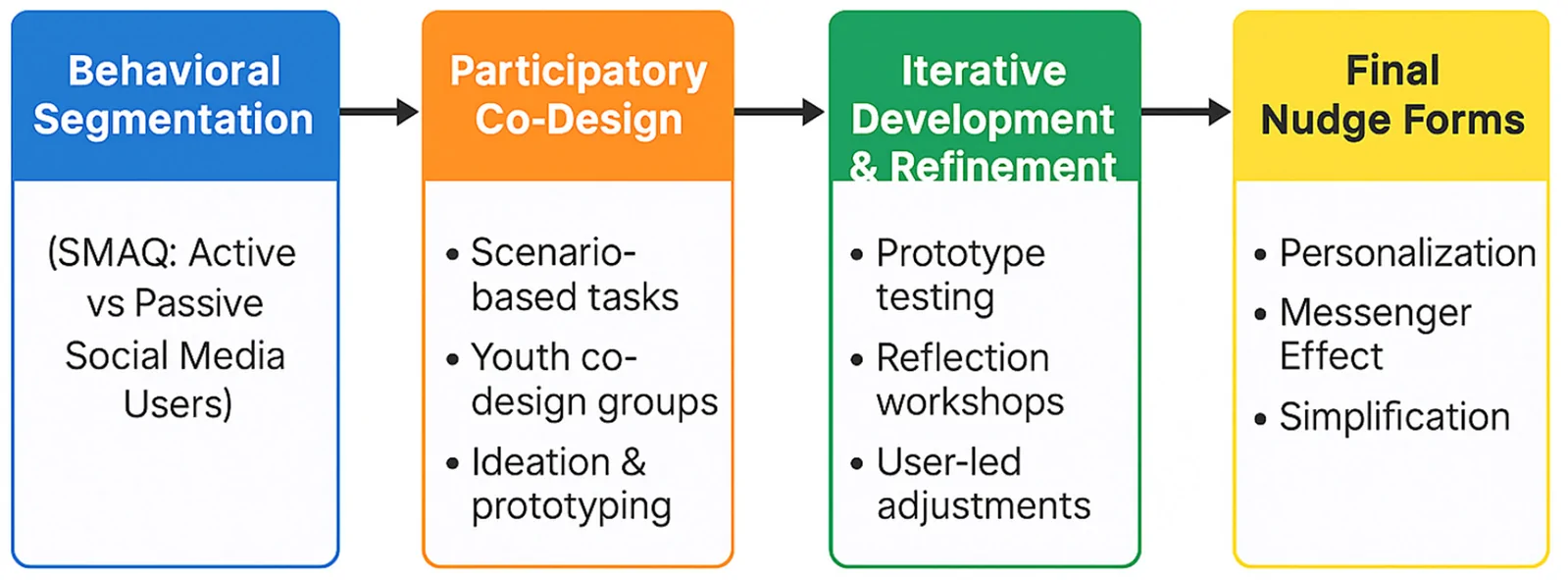
Overview of the co-design digital-nudging framework integrating behavioral segmentation, co-design workshops, and iterative refinement.

The study presents a user-friendly culturally based framework of digital-nudge intervention design to promote GESI among Arab youth as shown in Figure 2. This structure will involve a structured mixed-methods sequence approach: (i) the ethical approval and piloting process (Resnik, 2020); (ii) a quantitative profile of 32 participants through the Social Media Activity Questionnaire (SMAQ) disclosing a passive-engagement bias (weighted mean = 4.16 of story viewing vs. 2.09 of posting photos to family), which informed co-design group stratification (Ozimek et al., 2023); (iii) four interactive prototyping workshops, in which the participants developed 12 nudges. This interconnected workflow, that is, the behavioral segmentation, participatory design, and systematic refinement, is a culturally responsive and methodologically rigorous framework that can be applied to various sociocultural contexts. Although the framework draws conceptually from participatory design, the actual implementation follows a co-design approach, as participants actively contributed to ideation, prototyping, and refinement (States, 2022; Thaler and Sunstein, 2012; Cohen, 2008; Johnson et al., 2017).

### 3.2 Participants recruitment

Participants were recruited by personal networks and targeted e-mail distribution using targeted sampling. The criteria for inclusion included: (1) an independent Arab ethnic group, (2) an age between 18 and 24 years, and (3) a current residence in Qatar. All participants were provided with detailed information explaining the purposes, procedures and eligibility requirements of the study. Written informed consent was obtained before participating in the survey and the workshops in accordance with the institutional research ethics protocol. A total of 32 participants took part in both phases of data collection, encompassing the survey and the co-design workshops. The sample size (*n* = 32) is consistent with qualitative research guidelines recommending 20–30 participants for exploratory co-design studies to reach early thematic saturation and yield robust formative feedback (Guest et al., 2006). The study was approved by the Institutional Review Board (IRB) of Hamad Bin Khalifa University (HBKU-IRB-2024-30). All participants provided explicit informed consent, including for audio recording where applicable, and were assured of confidentiality, voluntary participation, and the right to withdraw. Data storage adhered to IRB security and governance guidelines, and all procedures followed ethical standards for research involving human subjects. Anonymized data and analysis scripts are available from the corresponding author upon reasonable request.

### 3.3 Pilot study

Pilot studies are essential for refining data collection procedures and addressing potential challenges before scaling up research (Ismail et al., 2018; Hassan et al., 2006). To ensure rigor, a detailed plan and a predefined timeline were established

and shared with participants in advance. The pilot study began with distributing invitations to potential participants. A total of ten individuals—including several HCI researchers—were subsequently recruited. This sample size aligns with pilot-study guidelines, which recommend small and diverse groups capable of providing rapid feedback and reaching early thematic saturation (Braun and Clarke, 2006). Prior to participation, all individuals signed electronic consent forms, in accordance with the Institutional Review Board approval obtained for this study (HBKU-IRB-2024-30).

The pilot assessed four key feasibility indicators: (i) recruitment and consent return rates, (ii) completion of the SurveyMonkey questionnaire, (iii) workshop logistics (including presentation flow, group work, and feedback), and (iv) identification of procedural bottlenecks. Success thresholds were predefined as achieving at least 80% consent return, 90% questionnaire completion, and a maximum of 30 min per design activity (Thabane et al., 2010). To test the survey and workshop procedures, participants first completed a brief SurveyMonkey questionnaire, selected for its accessibility and suitability for remote administration (Smith et al., 2023; Alibasic et al., 2022; Frey and Bloch, 2023). Following the survey phase, the session transitioned into a co-design workshop. The facilitator opened with an overview of digital nudges, integrating principles from behavioral science and Nudge Theory to explain different types of nudges and their practical applications (Al-Mansoori et al., 2023). A scenario-based presentation was then used to frame the problem context and emphasize the need for the proposed digital nudges (Turner, 1998; Dahl and Sharma, 2022).

Participants subsequently divided into three groups and collaboratively designed digital nudges. Upon completing the design activities, each group presented its outputs. These presentations created opportunities for constructive critique, reflection, and iterative refinement (Dahl and Sharma, 2022). The session concluded with a brief discussion and appreciation of participants' contributions. The research team then analyzed the collected data: SurveyMonkey responses were summarized using descriptive statistics, and workshop artifacts were examined through thematic analysis following (Braun and Clarke, 2006). This analysis guided refinements to the study design.

Several improvements were implemented in response to pilot feedback. First, gender diversity was strengthened by balancing invitations to ensure equal representation, thereby incorporating a broader range of perspectives (Bardzell, 2010; Dray and Siegel, 2014; Baer and Kaufman, 2008). Second, to reduce administrative delays, all consent forms were collected in advance through online submission. Third, Methodological enhancements were incorporated to strengthen participant preparation and optimize the overall structure of the workshop. Prior to the session, participants received pre-workshop materials and warm-up briefings designed to provide cognitive scaffolding and establish a shared conceptual foundation for the subsequent activities (Wood et al., 1976; Knapik, 2006; Thomas, 2021). The workshop presentation also integrated a concise multimedia component outlining the historical development and theoretical underpinnings of nudge theory. The use of video-based instructional material has been shown to facilitate the reinforcement of core concepts, promote deeper cognitive processing, and improve information retention (Shekhar, 2017; Kramer, 2023; Mudgal, 2023). To sustain engagement and ensure smooth transitions between activities, short scheduled breaks were incorporated throughout the session.

### 3.4 SMAQ questionnaire: active and passive social media behavior

The co-design workshop process, a collaborative endeavor, brings together diverse stakeholders to actively participate in designing solutions, products, or services (Denecke et al., 2023). To prepare for the workshops, each participant completed a survey designed to assess their social media engagement preferences and behaviors, specifically identifying whether they were passive or active users (Rule et al., 2020; Pearson et al., 2020). Distinguishing between active and passive social media users was essential because these behavioral patterns directly determine how individuals engage with online content, which in turn shapes the design of targeted digital nudges. Meta-analytic evidence shows that active behaviors (e.g., creating and interacting) and passive behaviors (e.g., scrolling and observing) lead to markedly different social and psychological outcomes, necessitating tailored intervention strategies (Godard and Holtzman, 2024).

The rapid increase in social media platforms has profoundly altered communication dynamics, reaching virtually every aspect of modern life (Rozgonjuk et al., 2020). As researchers increasingly explore social media usage and its societal impacts, the distinction between passive and active engagement has gained significant attention. Choosing an appropriate questionnaire instrument is challenging due to the numerous platforms and evolving digital trends (Rozgonjuk et al., 2020). It is essential to identify a tool that is comprehensive, valid, adaptable, and capable of capturing the subtle differences in passive and active engagement across various platforms (Ozimek et al., 2023; Hourihan, 2020; Fioravanti et al., 2021; Trunfio and Rossi, 2021). Recent research underscores the critical distinction between passive and active social media users in understanding engagement patterns. Foundational studies by Lampe et al. (2011) and Orosz et al. (2016) provide significant insights into Facebook usage motivations and user behaviors. However, gaps persist regarding the validity and depth of existing assessment tools, as highlighted by critiques in studies such as Ozimek et al. (2023). Recognizing the need for a more refined assessment tool, Ozimek and Bierhoff (2016) introduced the German Facebook Activity Questionnaire (FAQ), categorizing active (Acting), passive (Watching), and self-presentation (Impressing) behaviors. Subsequent research by Trifiro and Gerson (2019) further developed this framework, introducing a three-factor scale covering active social, active non-social, and passive use. However, these scales primarily focused on Facebook, leaving gaps in understanding user behavior on other platforms. With the rapid expansion of social media platforms, there emerged a need for a comprehensive assessment tool that goes beyond Facebook. While some studies developed scales for specific platforms like Instagram, a validated measure encompassing broader social media use was lacking. Thus, the introduction of the SMAQ aimed to address this gap. Their study recruited 1230 participants to assess the validity and reliability of the SMAQ. Both exploratory and confirmatory factor analyses confirmed the two-factor structure of SMAQ, effectively distinguishing between active and passive social media use. Internal consistency analyses highlighted the high reliability of both scales.

In our investigation, we employed the SMAQ questionnaire (Ozimek et al., 2023) to differentiate between passive and active social media users. The SMAQ questionnaire was selected because it offers a validated two-factor structure, demonstrates high reliability, and captures engagement across multiple platforms, making it suitable for profiling users in a rapidly evolving social-media ecosystem. The questionnaire begins with demographic inquiries regarding gender, self-identification, educational background, and field of study, providing contextual information crucial for subsequent analysis. This contextualization mirrors the study's focus on understanding distinct patterns of social media activity and engagement. Following the demographic section, participants indicate their frequency of engagement in various social media activities using a 5-point Likert scale. These activities range from passive actions such as viewing photo albums and reading others' profiles to more active behaviors like posting photos, creating events, or participating in discussions. By employing a Likert scale, the questionnaire captures nuanced differences in participants' social media engagement, allowing for the identification of both passive and active users. The questionnaire instrument was implemented online through the SurveyMonkey platform, facilitating anonymous responses used by many scholars (Maytin et al., 2021). The 5-point Likert format enables fine-grained differentiation in engagement frequency, allowing for precise segmentation of participants whose behavioral profiles informed subsequent workshop grouping and nudge prototyping (Ni et al., 2020).

#### 3.4.1 Statistical validation and reliability

Prior to the main analysis, data quality and reliability checks were conducted to ensure the robustness of the SMAQ measurements. Missing values were examined and addressed using listwise deletion, as fewer than 3% of responses were incomplete (Hair et al., 2019). Outliers were evaluated using standardized z-scores (|*z*|>3), and none were found to influence descriptive trends, reinforcing the integrity of the dataset. Skewness and kurtosis values fell within the recommended ±1 range, indicating approximate normality and supporting the use of parametric descriptive statistics such as means and standard deviations (Field, 2018).

Internal consistency was assessed using Cronbach's α, a standard metric of reliability in social and behavioral research (Tavakol and Dennick, 2011). The overall reliability coefficient was α = 0.82, well above the 0.70 benchmark typically recommended for exploratory work (Nunnally and Bernstein, 1978). This level of internal consistency confirms that the active- and passive-use sub scales are psycho-metrically sound and can be confidently used for subsequent subgroup analyses. Because the scales demonstrated adequate reliability, participants could be reliably segmented into active and passive user profiles, which informed the structuring of workshop activities and the development of targeted digital-nudge concepts in the following phase of the study. Weighted averages were computed using the total number of *valid* responses per activity as the denominator, with the instrument quality screening indicating fewer than 3% incomplete submissions. Per-activity sample counts are explicitly reported in the Results (e.g., *n* = 32 for fully completed items), ensuring computational transparency and reproducibility. We acknowledge that SMAQ self-reports may be influenced by social-desirability bias, particularly given the gender-sensitive context. Future work should complement self-report segmentation with behavioral usage logs or API-derived metrics to enable objective cross-validation (Braun and Clarke, 2006).

#### 3.4.2 Reproducibility

All descriptive and reliability analyses were performed using IBM SPSS Statistics (Version 28) (IBM Corp., Armonk, NY, USA) (IBM, 2021) and cross-validated in Python (Version 3.11) (Python Software Foundation, USA) using the pandas (Pandas Development Team T, 2020), SciPy (Virtanen et al., 2020), and NumPy (Harris et al., 2020) libraries to ensure computational accuracy. The anonymized dataset and analysis scripts are available from the corresponding author upon reasonable request, supporting transparency and reproducibility of findings. The validated behavioral profiles guided the co-design workshops and ensured the nudges aligned with participants' preferences.

### 3.5 Co-design workshops

Four co-design workshops were structured in person to facilitate seamless participant interaction, drawing on everyday modalities of communication such as face-to-face dialogue and shared artifacts, which have been shown to enhance trust and collaborative dynamics in participatory design contexts (Smith, 2020).

**Figure 3:**
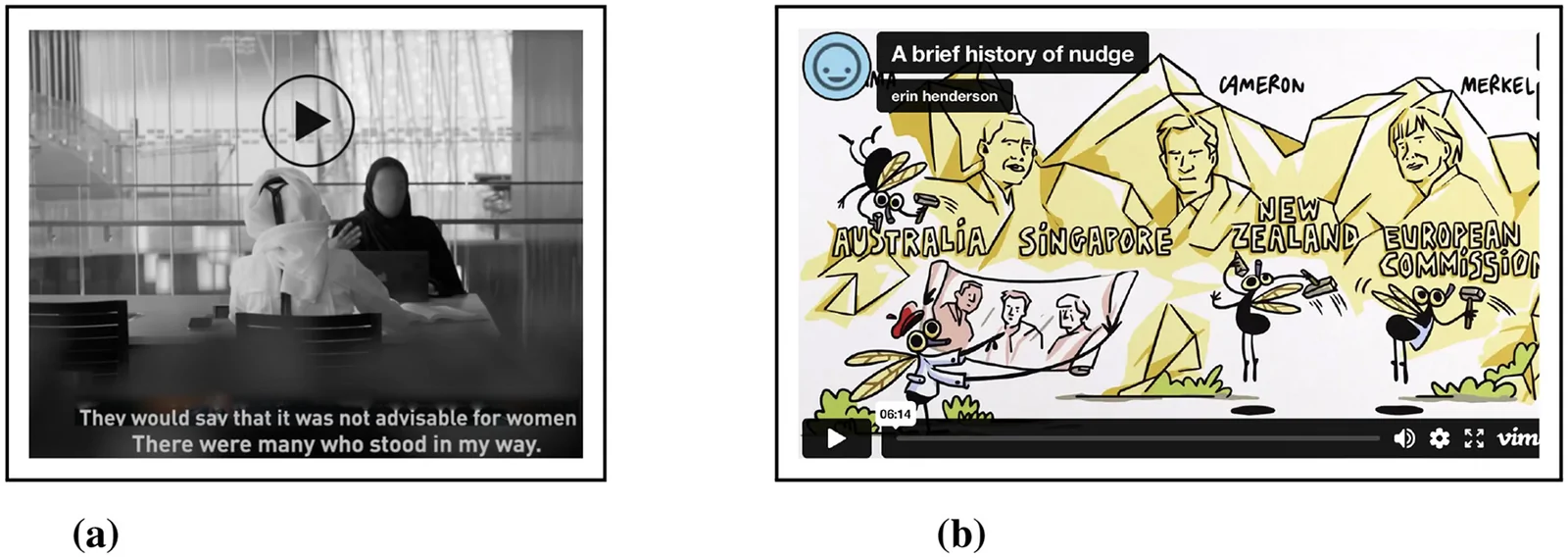
**(a)** Warm-up video used before the co-design workshops: Video on GESI in Qatar. **(b)**Warm-up video used before the co-design workshops: Video introducing nudge theory.

Three workshops were held at the Hamad Bin Khalifa University campus in a multimedia room equipped for hybrid lectures and seminars, while one took place in a café, pre-arranged with a data show and necessary tools. The locations were chosen based on participants' preferences, availability, and ease of access, forming 12 groups, despite initial challenges in recruitment due to scheduling conflicts and ensuring diversity within the group. To prepare participants for the design activities, a booklet detailing various digital nudge typologies was provided beforehand as shown in Figure 3.

This served as a cognitive tool to reduce mental load, enabling participants to focus on the creative process with essential background information readily available. Prior to the workshops, consent forms were distributed to ensure adherence to ethical guidelines (Jones and Taylor, 2019). At the start of each workshop, participants were welcomed warmly, and a collaborative tone was set through an icebreaker activity featuring a video on the history of economic behavior and the application of nudges in policymaking (Johnson and Johnson, 2009; Thaler and Sunstein, 2008a). This activity encouraged participants to share examples from their own experiences, fostering a discussion. The next phases of the workshops consisted of two main parts: theory and application. During the theoretical part, the facilitator presented an overview of digital nudges, integrating principles from behavioral science and Nudge Theory to explain different nudge types and their practical applications. In the application phase, participants were introduced to a scenario-based challenge designed to contextualize the problem and emphasize the relevance of their designs.

**Figure 4:**
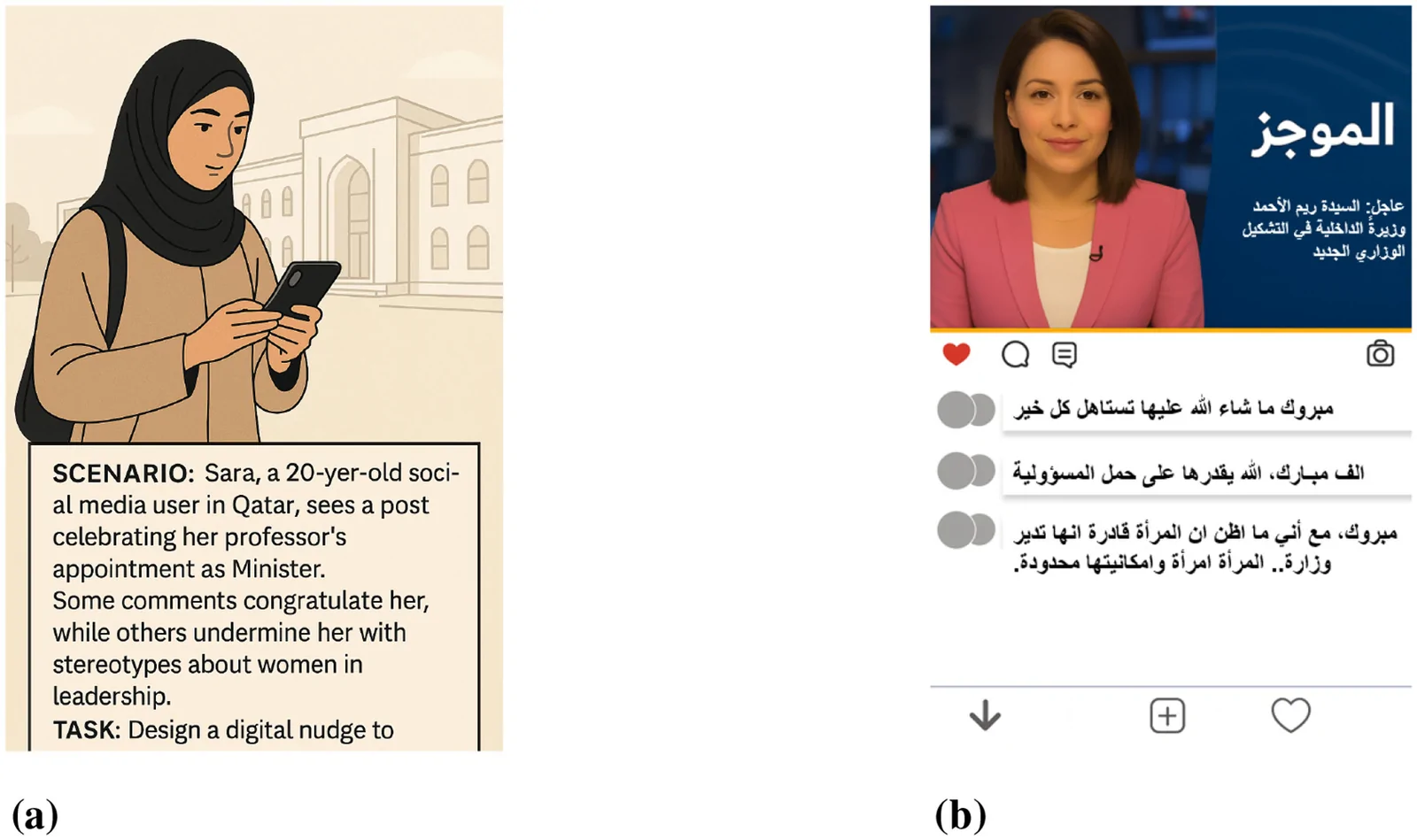
**(a)** AI-generated illustration of Sara, the central user in the scenario. **(b)** Social media post showing a news presenter announcing the appointment of a female minister in Qatar.

One scenario presented involved responding to online comments that undermined the appointment of a prominent female leader. The scenario featured Sara, a 20-year-old Arabic social media user, who encountered a social media post celebrating her professor's appointment as the new Minister of Communications in Qatar. While many congratulated the professor, some comments dismissed her qualifications and perpetuated harmful stereotypes about women in leadership. Figure 4a presents the AI-generated illustration of Sara, while Figure 4b shows the social media post she encountered. The post announces the appointment of a female minister in Qatar and includes mixed user comments: supportive messages such as “Congratulations, may God grant her success,” alongside a harmful stereotype stating “Some people say a woman's abilities are limited.” These materials provided the contextual stimulus for the co-design activity.

**Figure 5:**
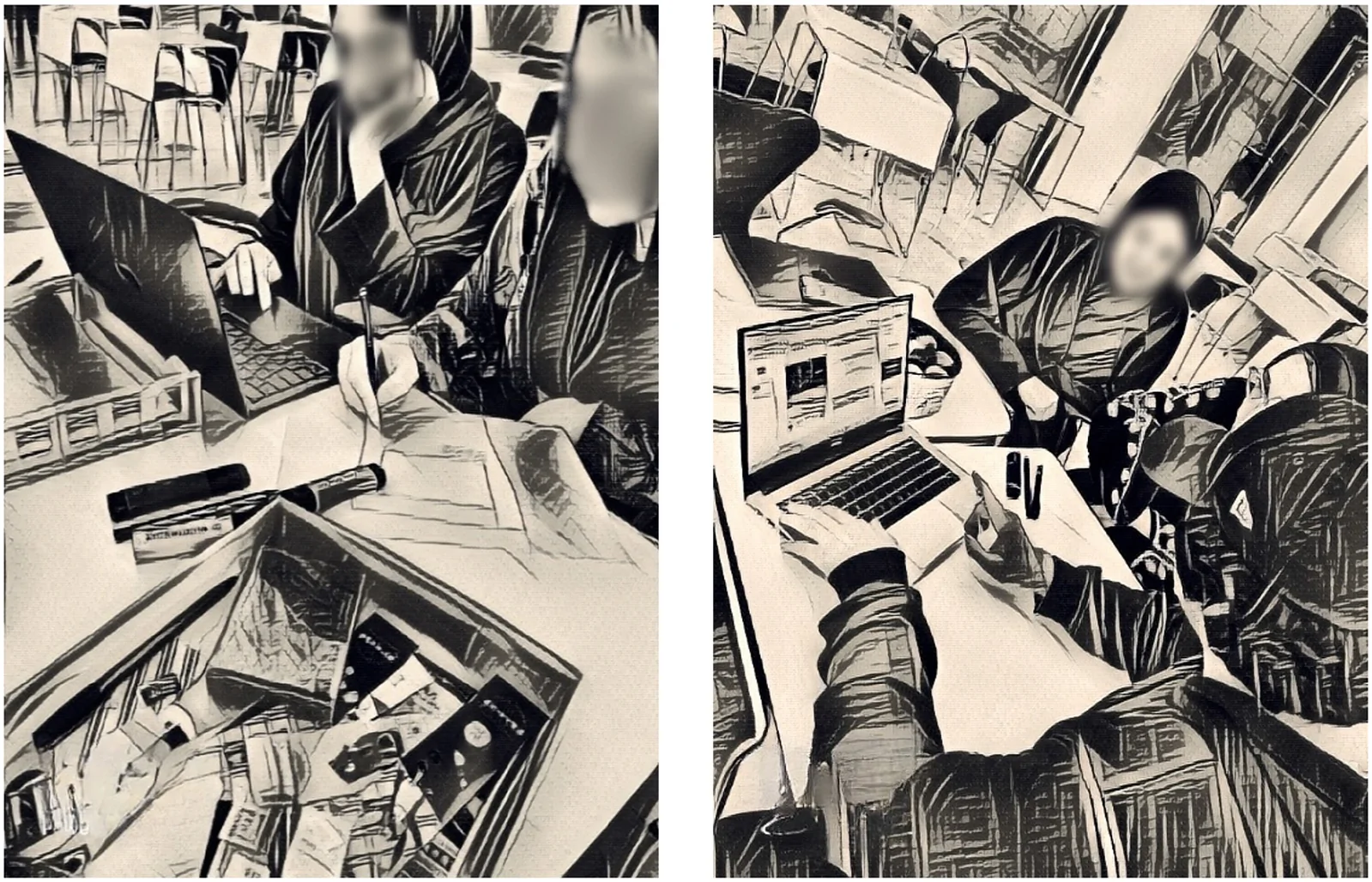
Participants collaboratively discussing and co-designing digital nudges during the workshop. They reviewed ideas, provided feedback to peers, and refined their nudge concepts to ensure alignment with their intended goals for promoting GESI in online communities.

Participants were tasked with designing digital nudges to promote positive discourse, challenge gender stereotypes, and foster inclusivity in online spaces. These constructs were reflected in participants' design choices and evaluated through the nature of proposed interactions and messaging strategies in the co-designed prototypes. Participants, divided into groups, collaboratively explored design directions using tools like Figma, Miro, and hand-drawn sketches, based on their skills and preferences (Brown and Smith, 2018). Through this hands-on exercise, participants co-created nudges aimed at encouraging respectful engagement, promoting GESI, and countering discriminatory comments online as shown in Figure 5.

At the end of each workshop, the groups presented their designs, which were refined iteratively based on feedback from other participants. The iterative process resulted in 12 final prototypes promoting GESI in online communities, focusing on three main types of nudges: personalization, messenger effect, and simplification. These prototypes were handed over to the development team for further implementation, marking the culmination of a collaborative and iterative design process (Smith, 2020; Jones and Taylor, 2019; Brown and Smith, 2018). It is worth mentioning that snacks were provided to maintain energy levels, and participants were awarded certificates of appreciation from the College of Science and Engineering at HBKU, recognizing their contributions.

#### 3.5.1 Reflection workshop

After the implementation phase, participants were invited to test the digital prototypes in a reflection session. Reflection sessions are critical for refining prototypes and ensuring that the final product meets the needs and expectations of its users (Benz et al., 2024). They help identify unforeseen issues and areas for improvement, making them invaluable for iterative design and development (König et al., 2022). The reflection session was divided into two parts based on participants' preferences and availability. The first session was conducted in person, with three participants attending, while the second part was conducted online, with fourteen participants attending. Both sessions involved participants verifying if the nudges matched what they had designed, allowing participants to review the nudges and provide feedback to ensure they met their needs and preferences.

#### 3.5.2 Workshops facilitator

The workshop was co-facilitated by the first author, whose prior engagement with participants helped build trust and familiarity. Consistent with McKercher's provocateur model, the facilitator intentionally encouraged critical reflection and challenged assumptions to mitigate groupthink and enhance the creativity of emerging design concepts (McKercher, 2020).

## 4 Results and discussion

### 4.1 Demographic results

**Figure 6:**
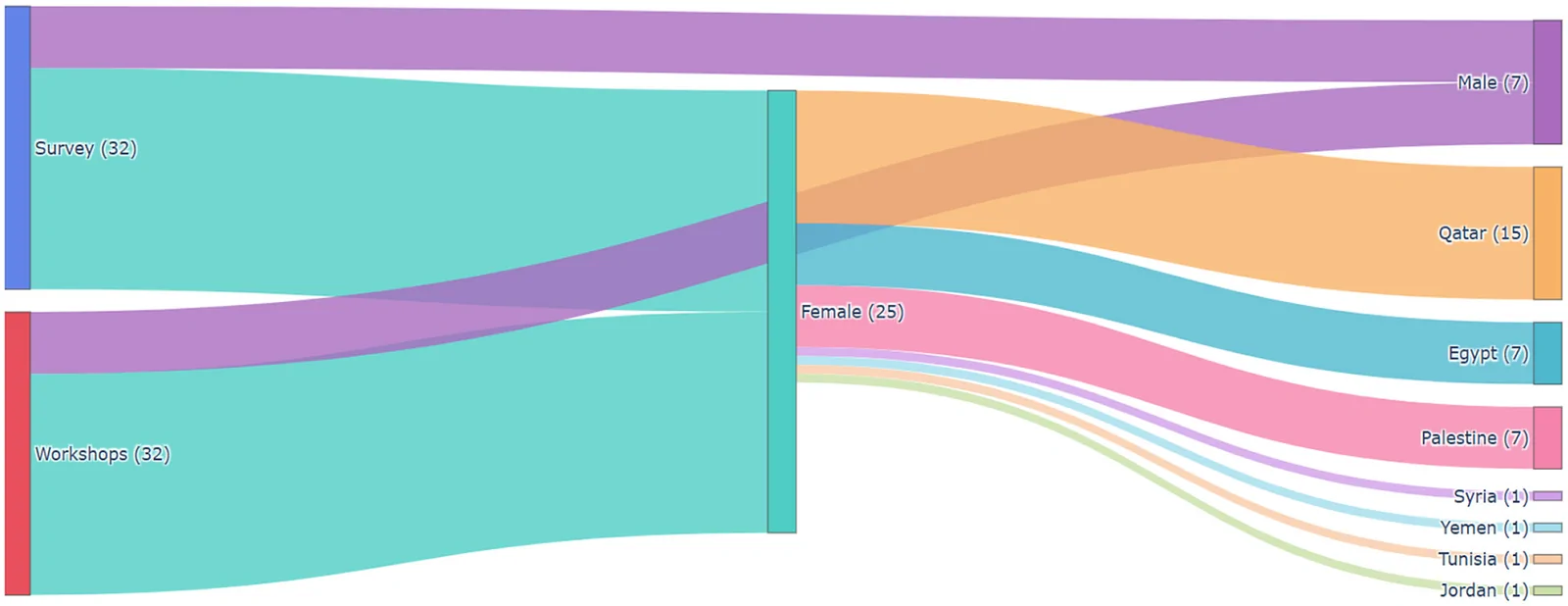
Sankey diagram showing participant distribution across co-design activities, gender, and countries. All 32 participants completed both Survey and Workshop stages. The sample included 25 females and 7 males Country representation was led by Qatar (15 participants), followed by Egypt (7) and Palestine (7), with smaller groups from Syria (1), Yemen (1), Tunisia (1), and Jordan (1). Flow widths are proportional to participant counts.

A total of 32 participants took part in the co-design activities, which included surveys and workshops. It is important to note that the sample included a higher proportion of female participants (78%) compared to male participants (22%), reflecting voluntary participation patterns within the study context. Regarding activity engagement, surveys and workshops each had 32 participants. These results suggest a high level of involvement in the co-design activities, with a strong preference for interactive and collaborative activities. The participant sample consisted of 32 individuals, with 25 females (78%) and 7 males (22%). The country distribution was diverse, with the majority of participants (15) from Qatar, followed by 7 participants each from Egypt and Palestine. Smaller groups included participants from Syria, Yemen, Tunisia, and Jordan, with one participant from each of these countries. Participants' ages ranged from 18 to 23 years, with the majority falling between 19 and 22 years. The most represented age group was 20 years old (7 participants), followed by 22 years old (6 participants) and 19 years old (5 participants). The 23-year-old group had the smallest representation, with only 2 participants. Figure 6 shows the overview of participants in co-design activities and their demographics, including the distribution of activities, gender, country, and age.

### 4.2 Quantitative results: measuring passive and active social media users

The study begins by establishing a quantitative baseline of social-media engagement among Arab youth. The participant sample included 32 youth aged 18–23, representing diverse national backgrounds while residing in Qatar. The quantitative analysis revealed a clear imbalance in digital interaction behaviors, where passive content consumption strongly outweighed active participation and content creation, echoing prior findings on lurking and peripheral participation in online communities (Nonnecke and Preece, 2001; Edelmann, 2013; Sunstein, 2014). For example, passive users inspired the design of low-effort interaction nudges (e.g., simplified responses), whereas active users influenced the inclusion of expressive and personalized engagement features. Presenting these findings upfront creates a crucial empirical foundation, allowing the study to justify and structure its co-design phase with precision (Sanders and Stappers, 2008). The identified behavioral gap directly informed the focus of the co-design workshops, in which participants were guided to generate nudge concepts that respond specifically to the previously observed interaction asymmetries (Thaler and Sunstein, 2008a; Meske and Potthoff, 2017). This approach ensures that all nudging strategies remain data-anchored, context-appropriate, and user-aligned from conceptualization to prototype evaluation.

The SMAQ survey data reveals distinct patterns of active and passive engagement based on the weighted average scores of various social media behaviors.

#### 4.2.1 Weighted average calculation

The weighted average is calculated using the formula:


$$\text{Weighted Average}=\frac{\begin{array}{c} \sum (\text{Percentage of responses}\times \;\\ \text{Corresponding score}) \end{array}}{\text{Total number of responses}}$$

Each response category—“*Very Unlikely,” “Unlikely,” “Neutral,” “Likely,” and “Very Likely”*—was assigned a corresponding numerical score (1, 2, 3, 4, 5, respectively). The percentage of respondents for each category was then multiplied by its respective score. The sum of these products was divided by the total number of responses to derive the weighted average for each activity.

For example, for the activity “*I post photos with my family,”* the weighted average was calculated as follows:


$$\text{Weighted Average}=\frac{\begin{array}{c} \left(53.13\%\times 1\right)+\left(18.75\%\times 2\right)+\;\\ \left(6.25\%\times 3\right)\\ +\;\left(9.38\%\times 4\right)+\left(12.50\%\times 5\right) \end{array}}{32}=2.09$$

This process was repeated for all activities to determine the weighted averages reflecting the relative engagement levels across various social media behaviors. The SMAQ survey data reveals distinct patterns of active and passive engagement based on the weighted average scores of various social media behaviors. The activities with the lowest weighted average scores, indicating lower likelihoods of engagement, are as follows:

- *I post photos with my family* (2.09)
- *I post links or video clips myself* (2.28)
- *I create events or invite users to events* (2.50)

These results suggest that participants are less likely to engage in active content creation, with limited sharing of personal or multimedia content and minimal involvement in event organization.

On the other hand, activities with higher weighted average scores, reflecting stronger likelihoods of engagement, include:

- *I look at the stories of my friends/my subscriptions* (4.16)
- *I read the comments on my own pictures* (4.09)
- *I read private messages that other users send me* (4.06)

These findings indicate a preference for passive consumption behaviors, such as viewing others' stories and reading personal messages or comments. Additionally, some activities exhibit more mixed engagement patterns, with moderate scores suggesting a blend of active and passive usage:

- *I look at the profiles/pages of other users or read through them* (3.41)
- *I respond to event invitations* (3.00)

The detailed distribution of responses for each activity, presented in Table 1, underscores a dominant preference for passive consumption over active content creation.

**Table 1:** Survey responses and weighted averages.

Activity	Very unlikely	Unlikely	Neutral	Likely	Very likely	Total	Weighted average
I post photos with my family	53.13%	18.75%	6.25%	9.38%	12.50%	32	2.09
I post links or video clips	40.63%	28.13%	6.25%	12.50%	12.50%	32	2.28
I create events or invite users to events	34.38%	15.63%	28.13%	9.38%	12.50%	32	2.50
I post photos	25.00%	15.63%	21.88%	25.00%	12.50%	32	2.84
I create groups	25.00%	12.50%	21.88%	28.13%	12.50%	32	2.91
I respond to event invitations	25.00%	6.25%	25.00%	31.25%	12.50%	32	3.00
I create highlights	18.75%	9.38%	28.13%	18.75%	25.00%	32	3.22
I look at the photo albums of other users	6.25%	15.63%	21.88%	43.75%	12.50%	32	3.41
I look at the profiles/pages of other users	6.25%	12.50%	34.38%	28.13%	18.75%	32	3.41
I read entries on the chronicles of others	6.25%	9.38%	31.25%	40.63%	12.50%	32	3.44
I read comments on other users' pictures	3.13%	15.63%	28.13%	37.50%	15.63%	32	3.47
I look at the profiles of my relatives	3.13%	12.50%	15.63%	50.00%	18.75%	32	3.69
I look at the “newsfeed” for latest activities	6.25%	3.13%	28.13%	34.38%	28.13%	32	3.75
I read private messages sent to me	9.38%	0.00%	15.63%	25.00%	50.00%	32	4.06
I read comments on my own pictures	6.25%	3.13%	12.50%	31.25%	46.88%	32	4.09
I look at the stories of friends/subscriptions	3.13%	3.13%	15.63%	31.25%	46.88%	32	4.16

Notably, passive social media behaviors, such as viewing stories from friends or subscriptions (mean: 4.16, standard deviation: 1.00) and reading private messages (mean: 4.06, standard deviation: 1.22), are highly prevalent among users. This is further reflected in the relatively high engagement with comments on personal photos (mean: 4.09, standard deviation: 1.13) and frequent checks of the newsfeed (mean: 3.75, standard deviation: 1.09). In contrast, active participation in content creation, such as posting links, video clips, or family photos, is notably lower, with mean scores of 2.28 and 2.09, respectively. These findings suggest that while users are more inclined to engage in passive social media behaviors, they exhibit a reluctance toward generating and sharing personal content, particularly those involving family-related photos. Furthermore, the standard deviations indicate significant variability in certain activities, such as reading private messages, where some users demonstrate high engagement while others remain less active. Table 2 summarizes the mean, median, standard deviation, and range of responses for these activities, highlighting that passive activities such as reading messages or viewing newsfeeds tend to have higher engagement levels compared to more active behaviors like posting photos or creating events.

**Table 2:** Mean, median, standard deviation, and range for various social media activities.

Activity	Min	Max	Median	Mean	Std. Dev.
I look at the photo albums of other users	1.00	5.00	4.00	3.41	1.09
I look at the profiles/pages of other users or read through them	1.00	5.00	3.00	3.41	1.11
I look at the stories of my friends/my subscriptions	1.00	5.00	4.00	4.16	1.00
I read private messages that other users send me	1.00	5.00	4.50	4.06	1.22
I read entries on the chronicles and personal pages of other users	1.00	5.00	4.00	3.44	1.03
I read through the comments on other users' pictures	1.00	5.00	4.00	3.47	1.03
I read the comments on my own pictures	1.00	5.00	4.00	4.09	1.13
I look at the profile pages of my relatives	1.00	5.00	4.00	3.69	1.01
I look at the “newsfeed” to see the latest activities of other users	1.00	5.00	4.00	3.75	1.09
I create events or invite users to event	1.00	5.00	2.50	2.50	1.37
I post links or video clips myself	1.00	5.00	2.00	2.28	1.42
I post photos with my family	1.00	5.00	1.00	2.09	1.44
I create groups	1.00	5.00	3.00	2.91	1.38
I post photos	1.00	5.00	3.00	2.84	1.37
I respond to event invitations	1.00	5.00	3.00	3.00	1.37
I create highlights	1.00	5.00	3.00	3.22	1.41

As shown in Table 2, participants reported higher mean scores for passive engagement behaviors (e.g., viewing and liking posts) compared to active forms (e.g., sharing or commenting), consistent with the weighted-average patterns presented earlier. This indicates that youth participants primarily engage as observers rather than as active contributors on social platforms. The trend aligns with previous findings on “lurking” participation behavior in online communities (Ellison et al., 2007).

#### 4.2.2 Inferential insights

Due to the limited sample size (*n* = 32), inferential testing was not pursued to avoid over interpretation or inflated error rates. Instead, the analysis focused on descriptive trends supported by effect-size estimates, which provide insight into the relative strength of observed patterns without relying on statistical significance. This approach aligns with best practices for exploratory, small-sample behavioral studies (Cohen, 1988; Field, 2018). The result effect-size measures helped interpret the practical importance of the differences observed across active and passive engagement behaviors, supporting transparent and contextually grounded reporting.

#### 4.2.3 Sample size and representativeness

The final quantitative dataset comprised 32 valid responses. Although modest in size, this sample is appropriate for exploratory mixed-method research aimed at identifying patterns rather than testing hypotheses (Guest et al., 2020). Participants were purposively recruited to ensure diversity across gender, educational background, and university affiliation, reflecting the heterogeneity of Arab youth in Qatar. Nonetheless, the limited number of responses constrains the generalizability of the results and underscores the importance of conducting larger-scale replications in future studies to validate these preliminary insights.

### 4.3 Qualitative results: co-design workshops

Across the four workshops, participants acted as co-designers, collectively shaping and refining digital-nudge concepts that embodied their own values and everyday online practices (Chan et al., 2025; Shin, 2024). This participatory process generated twelve prototypes aimed at promoting GESI in online communities. A nudge—defined as a subtle modification to the choice environment that alters people's behavior in predictable ways without restricting options or changing economic incentives (Thaler and Sunstein, 2008b)—provided the foundational lens through which these prototypes were developed. In reflecting on how such behavioral cues operate within culturally grounded digital environments, participants produced artifacts that converged into three overarching nudge forms: simplification, messenger effects, and personalized/humor-based prompts.

**Figure 7:**
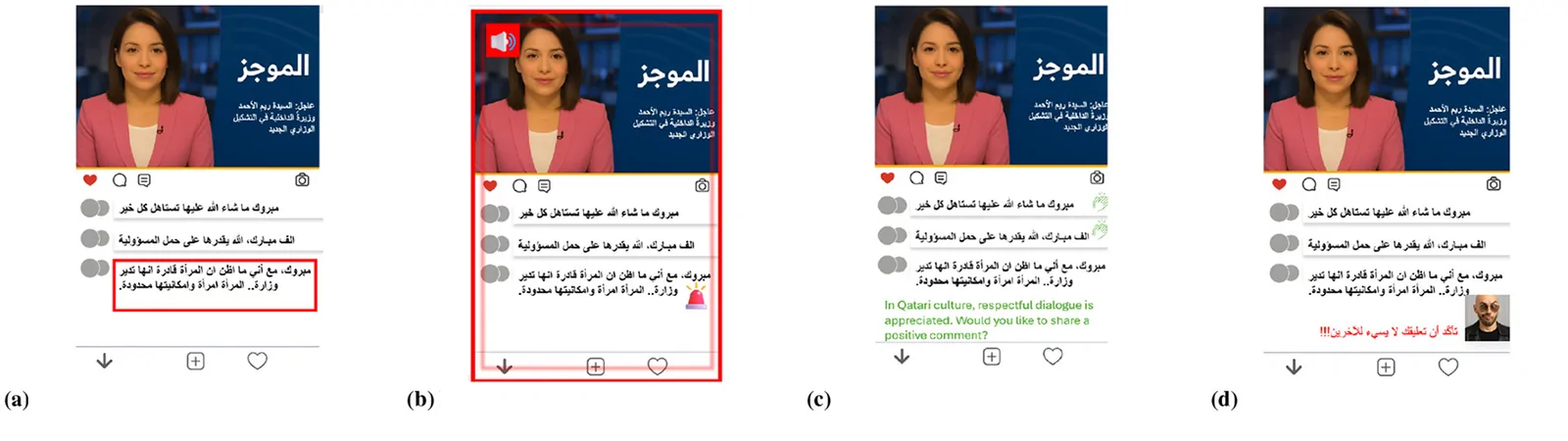
Representative digital-nudge prototypes co-designed by participants to promote Gender Equality and Social Inclusion (GESI) in online communities. **(a)** Simplification. The red highlight isolates the gender-biased comment. **(b)** Messenger effect (variant). A red-alert icon flags the biased comment. **(c)** Messenger effect. A culturally framed cue promotes engagement. **(d)** A personalized/humor nudge uses a familiar avatar.

The simplification form streamlined complex comment threads by visually isolating harmful or biased remarks, reducing cognitive load and helping users focus on GESI-supportive content (Brown et al., 2024). The messenger effect emerged in two culturally meaningful variants: a system-authority cue using warning icons to signal institutional disapproval, and a culturally grounded cue invoking Qatari norms of respectful dialogue to enhance credibility and social acceptance (Sunstein, 2015; The Decision Lab, n.d.). The personalized/humor form leveraged a familiar social-media avatar and light satire to diffuse negativity and motivate supportive replies (Mills, 2020; Sharma et al., 2025). Together, these prototypes demonstrate how co-designers adapted behavioral-economics principles to their cultural and digital realities. Figure 7 and Table 3 present representative examples of these nudge forms, reflecting the four designs most preferred by participants based on voting.

**Table 3:** Summary of representative co-designed digital nudge prototypes.

Prototype	Nudge type	Behavioral mechanism	Intended outcome
Simplification prototype	Simplification	Visually isolates harmful or biased comments to reduce cognitive load and direct users' attention toward problematic content.	Helps users identify discriminatory remarks more quickly and respond with lower interaction effort.
Warning-icon prototype	Messenger effect (system-authority variant)	Uses a red-alert cue to signal institutional disapproval and increase the salience of harmful comments.	Discourages biased commenting and prompts reconsideration of harmful language.
Culturally framed cue prototype	Messenger effect (culturally grounded variant)	Invokes culturally familiar norms of respectful dialogue to enhance credibility and relevance.	Encourages respectful engagement and culturally aligned support for GESI.
Avatar-based humorous prompt	Personalized/humor-based prompt	Uses a familiar avatar and light humor to personalize interaction and reduce defensiveness.	Increases positive engagement and encourages constructive responses.

A thematic analysis, guided by Braun and Clarke (2006), was conducted on the audio- and video-recorded data from all co-design workshop groups. This approach enabled systematic examination of how participants progressed through stages of planning, conceptualization, prototyping, and engagement with contextual factors, thereby yielding a process-based understanding consistent with established co-design methodologies.

Initially, researchers developed codes (sub-themes) content, capturing key aspects such as perceptions of gender equality, perceptions of social inclusion, and engagement with social media. Through iterative code generation, similar patterns across datasets were categorized per (Braun and Clarke, 2006) guidelines. Additionally, weekly meetings among the researchers were integral for refining the coding system and ensuring consistency and accuracy in identifying main themes. The analysis revealed important insights into the various stages of co-design, such as planning, conceptualization, prototyping, and the influence of contextual factors. The theme of planning emphasized the importance of task distribution and scheduling. Participants recognized the value of leveraging each individual's strengths, ensuring that roles were allocated based on expertise, as reflected in the quote, “Who's good at drawing? We may assign tasks based on what everyone's best at” (G2P3). This approach helped enhance the collaborative nature of the workshops, where all participants contributed meaningfully to the design process. Task distribution aligns with Sanders and Stappers (2008) perspective that users' lived experiences are crucial in shaping design outcomes. Additionally, scheduling was highlighted as an essential element to ensure that the workshop stayed on track. The importance of time management within co-design processes is reflected in the statement, “Alright, we've got the first 30 min to wrap up the nudge, after that, we'll move on to presenting the design” (G1P1). These insights suggest that structured planning can facilitate more efficient co-design workshops by encouraging participant engagement and enhancing the overall workflow. The Conceptualization theme revealed key activities involved in ideation, selection, and evaluation. Participants generated ideas freely, with one participant proposing, “What if we put a big red flag right in the middle of the screen?” (G6P2). This demonstrates the brainstorming phase, where the group engaged in creative thinking, laying the foundation for the design. However, as the process progressed, the group had to make decisions on which ideas to prioritize. This was particularly evident when participants deliberated on selecting ideas: “It's tough to decide—this one displays the warning message, and the other shows the notification message. Maybe we should just use both?” (G6P2). Evaluating ideas for feasibility, as discussed in the quote, “We need to make sure these ideas are doable before moving forward. Maybe we could use PowerPoint to lay them out and see how they look?” (G6P2), was also a crucial step in ensuring that the prototypes could be developed effectively. This iterative decision-making process, combined with the idea of prototyping development, reflects the evolving nature of co-design, where ideas continuously shift toward tangible solutions, as participants work together to create visual representations of their concepts. The Prototyping theme highlighted the iterative process of refining ideas through repeated cycles of development and feedback. The concept of Iterative Prototyping is central to this phase, where early concepts are continuously refined into more robust models. One participant's suggestion, “How about we add two lines of text here and shift the nudge over to this spot?” (G2P3), showcases how feedback from participants was incorporated into the evolving design. The iterative nature of prototyping allows for constant refinement and testing, ensuring that the final product aligns with the users' goals and expectations. Finally, the theme of Contextual Factors highlighted the role of context mapping, making exercises, and feedback gathering in shaping the final design. The importance of understanding the context within which the design will be implemented was emphasized in the statement, “... so, as you noticed in the video and presentation, our designs aim to promote GESI in online comments on social media,” as the facilitator explained. This contextual understanding is crucial in ensuring that the design solutions are relevant to the users' lived experiences. Additionally, feedback from other stakeholders, as exemplified in the quote, “I think if I came across your design, it wouldn't nudge me to write a positive comment supporting gender equality or social inclusion. I feel adding a more warning tone would be more effective” (G6P3), was used to refine and improve the prototypes. Feedback shows that design for GESI nudges needs continuous updates to stay relevant and connect with the audience (Table 4).

**Table 4:** Themes, sub-themes, descriptions, and representative quotes from co-design workshops.

Theme	Sub-theme	Description	Quotes
Planning	Task distribution	In co-design, tasks, and responsibilities are distributed among participants to leverage their unique expertise and ensure a collaborative and efficient design process. When users are considered “experts of their experiences,” their insights and feedback play a crucial role in shaping the design outcomes (Sanders and Stappers, 2008).	“Who's good at drawing? We may assign tasks based on what everyone's best at.” (G2P3)
Planning	Scheduling	The process of allocating specific time slots for distinct tasks or activities within a project or workshop (White et al., 2023).	“Alright, we've got the first 30 min to wrap up the nudge, after that, we'll move on to presenting the design.” (G1P1)
Conceptualization	Ideation	Conceptualization or concept generation is one of the design process's early and most important phases (Pan et al., 2010). Concepts serve as the foundation for further development and exploration in the design process (Sanders and Stappers, 2008).	“I've got an idea—what if we put a big red flag right in the middle of the screen?” (G6P2)
Conceptualization	Selection	The process of evaluating and choosing the most promising ideas from a pool of generated ideas (Baruah et al., 2021).	“It's tough to decide—this one displays the warning message, and the other shows the notification message. Maybe we should just use both?” (G6P2)
Conceptualization	Evaluation	The assessment of selected ideas to determine their feasibility for development ensures that only the most viable ideas move forward (Baruah et al., 2021).	“We need to make sure these ideas are doable before moving forward. Maybe we could use PowerPoint to lay them out and see how they look?” (G6P2)
Prototyping	Prototyping development	A prototype is a tangible representation of an idea. It can be a physical model, a digital simulation, or any other form that allows designers and users to interact with and test the idea (Sanders and Stappers, 2008).	“I'm not the best at drawing, but I'll give it a shot to help visualize what we're thinking.” (G7P4)
Prototyping	Iterative prototyping	This process is like “growing” early concepts into more mature products (or services, environments, experiences, etc.) through continuous prototyping (Sanders and Stappers, 2014).	“How about we add two lines of text here and shift the nudge over to this spot?” (G2P3)
Contextual factors	Context mapping	The process of gathering and using information about context to help create solutions for design teams (Visser, 2005).	“...so, as you noticed in the video and presentation, our designs aim to promote gender equality and social inclusion in online comments on social media,” the facilitator.
Contextual factors	Making exercise	A generative activity where participants use various materials and tools to create tangible representations of their thoughts, feelings, or experiences related to a specific topic (Visser, 2005).	“...as you saw in the scenario presented earlier, Sara, a 20-year-old, faced comments against gender equality and social inclusion... Now, we will form groups to design a nudge as part of our tasks,” the facilitator.
Contextual factors	Group presentation	Communicating and presenting the designs involves creating a narrative that conveys participants' experiences, needs, and contexts effectively (Visser, 2005).	“Our nudge is all about tackling negative comments by highlighting them in red and showing this image. We use some funny celebrity influencers and a short message to push for positive vibes. We threw in a funny way to keep it cool and grab attention.” (G8P3)
Feedback	Feedback gathering	The process of gathering and incorporating other stakeholders' feedback to refine and improve design prototypes (Sanders and Stappers, 2014).	“I think if I came across your design, it wouldn't nudge me to write a positive comment supporting gender equality or social inclusion. I feel adding a more warning tone would be more effective.” (G6P3)

The co-design workshops demonstrated the importance of collaboration and structured planning in generating effective solutions for promoting GESI through digital nudges. Participants actively contributed to the design process, with tasks being distributed based on individuals' strengths, which enhanced engagement and ensured that everyone played a meaningful role. The structured approach to task distribution aligns with the idea that users' lived experiences are crucial in shaping the outcomes of co-design activities. During the conceptualization phase, participants actively brainstormed and evaluated ideas, demonstrating creativity and a willingness to experiment. The selection and evaluation of ideas showcased the iterative nature of the process, where participants weighed the feasibility of different designs and refined them to ensure they could be practically implemented. This step aligns with the core principles of co-design, where feedback and discussion shape the design toward more feasible and impactful solutions.

The mixed-methods design revealed a clear behavioral asymmetry among Arab youth in Qatar: passive consumption of social-media content was dominant, while active creation (e.g., posting photos, videos, or events) was infrequent. This pattern aligns with prior observations of “lurking” behavior in online communities, where users derive social value from observation rather than contribution (Bennett and Segerberg, 2012; Ellison et al., 2007). The weighted-average scores (e.g., 4.16 for viewing stories vs. 2.09 for posting family photos) underscore the cultural and normative constraints that shape digital expression in the region. Co-Design workshops demonstrated that participatory involvement can surface culturally resonant nudge concepts. Participants repeatedly emphasized transparency, cultural sensitivity, and messenger credibility—elements identified as critical for ethical digital nudging (Schmidt and Engelen, 2020; Kuyer and Gordijn, 2023). The iterative prototyping process, supported by task distribution and real-time feedback, produced twelve prototypes that integrated messenger effects, personalization, and simplification. These design choices echo the Digital Nudging Process Model (DINU) and Human-Centered Design principles, yet extend them by embedding local gender norms and social-inclusion values, thereby addressing a noted gap in Western-centric nudge literature. The thematic analysis of workshop discourse highlighted four interrelated themes: planning (task allocation and scheduling), conceptualization (ideation, selection, evaluation), prototyping (iterative refinement), and contextual factors (context mapping, feedback). Together, these stages facilitated a sense of ownership among participants and mitigated ethical concerns around autonomy and manipulation, consistent with Co-Design theory that stresses user agency (Sanders and Stappers, 2008). Limitations include the modest sample size (*n* = 32) and the reliance on self-reported measures, which constrain generalizability. Future research should scale the framework, test prototype efficacy through controlled experiments, and explore longitudinal impacts on gender-equality attitudes. By combining culturally grounded Co-Design with evidence-based digital nudging, this study offers a replicable pathway for fostering inclusive online behavior in non-Western contexts. Building on the findings, this study proposes a unified framework linking user characteristics, behavioral tendencies, nudge mechanisms, and GESI-related outcomes. Specifically, passive users are more responsive to simplification-based nudges that reduce cognitive and interaction effort, while active users benefit from personalization and messenger-effect nudges that enhance relevance and trust. These nudge mechanisms, when aligned with users' behavioral profiles, contribute to increased participation, more constructive discourse, and greater inclusivity in online environments. This integrated perspective highlights how co-designed digital nudges can effectively translate behavioral insights into context-sensitive interventions that promote GESI.

## 5 Limitations and future work

Despite these contributions, several limitations should be acknowledged. Reliance on self-reported data through the SMAQ questionnaire introduces potential social-desirability bias, particularly in gender-sensitive contexts. Although efforts were made to balance gender representation, female participants were more numerous, which may have influenced the perspectives emphasized in the co-design outputs. In addition, the modest sample size (*n* = 32) constrains generalizability, and the single institutional setting may have shaped participant dynamics. Addressing these limitations will require complementing survey-based segmentation with objective behavioral logs, expanding co-design activities to larger and more diverse samples across Arab societies, and testing prototypes in real-world digital platforms. Future work will therefore focus on evaluating these nudges through longitudinal deployment and empirical assessment of their effectiveness in sustaining inclusive online engagement. Methodological enhancements will include purposive stratification to balance gender, nationality, and digital-literacy levels, as well as the use of think-aloud protocols during prototype interaction to capture real-time decision mechanisms, with reconfirmed consent for audio recording to ensure ethical compliance. These steps will advance culturally responsive digital-nudge frameworks that promote GESI in everyday digital interactions.

## 6 Conclusion

This study presented the full design, implementation and mixed-method analysis of a co-designed digital-nudging framework aimed at promoting GESI among Arab youth in Qatar. By integrating quantitative segmentation of active and passive social media behaviors with qualitative co-design workshops, the research demonstrated that culturally grounded digital nudges can translate GESI principles into actionable online practices. The twelve prototypes generated across the workshops converged into three nudge forms—*personalization, messenger effect*, and *simplification*—reflecting participant-led priorities for transparency, cultural sensitivity, and usability.

## Data Availability

The raw data supporting the conclusions of this article will be made available by the authors, without undue reservation.
