# Supplementary material for: Co-designing digital nudges to promote gender equality and social inclusion among Arab youth: a mixed-methods study
Source: Front Psychol. 2026 Sep 8;17:1763151. doi: 10.3389/fpsyg.2026.1763151 (PMC13593436; doi:10.3389/fpsyg.2026.1763151)

Understanding Digital Nudges: Forms and defintions

The Future of Digital Citizenship in Qatar: A Socio-Technical Approach

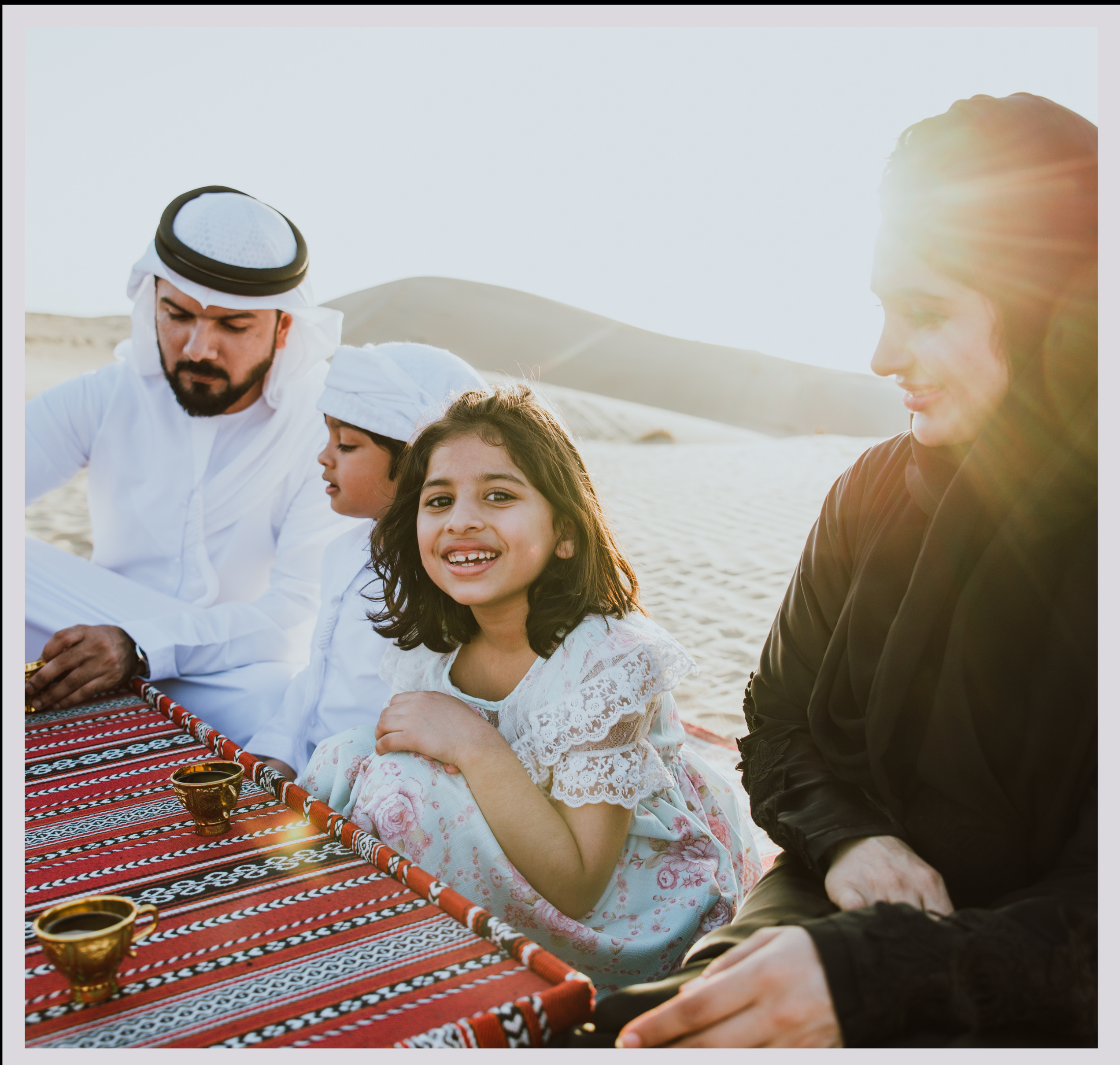

Together for a better Qatar

Introduction

Dugital Nudge : is a subtle form of using design, information and interaction elements to guide user behavior in digital environments, without restricting the individual’s freedom of choice

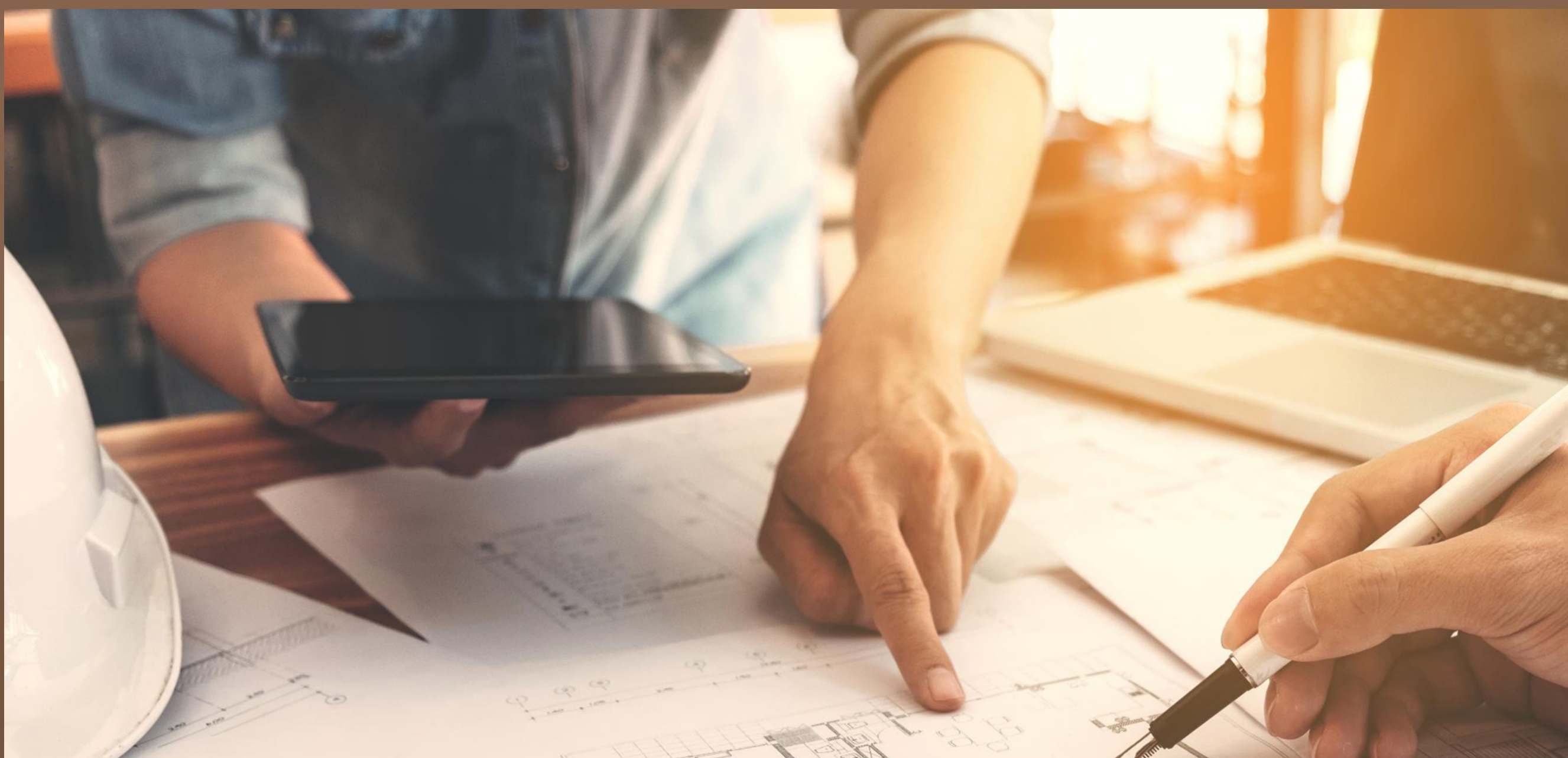

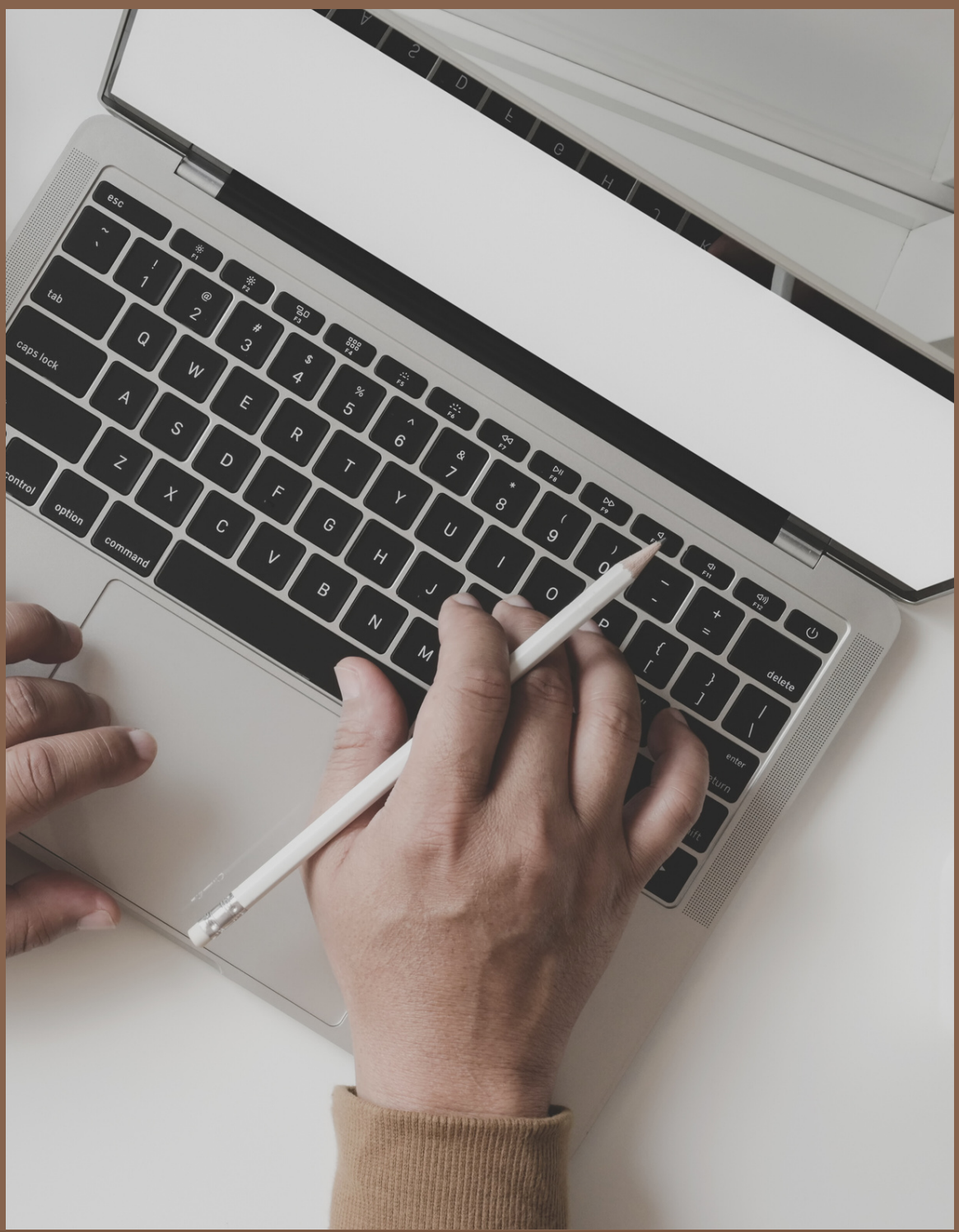

Feedback Nudges

Use a speech bubble or a star rating symbol to illustrate the importance of personalized feedback in guiding user behavior.(1)

Types of Digital Nudges

Social Proof Nudges

Display social media icons or thumbs-up symbols to represent the impact of social influence on decision-making.(2)

Show Reminders and Prompts

a clock or a notification bell to symbolize the effectiveness of timely reminders and prompts in encouraging action (3)

Personalization Nudges

Tailoring information or options to align with users' individual preferences or characteristics(4)

Forms of Digital Nudges

Emoji symbols

use emojis to influence user behavior in digital platforms. They leverage the visual impact of emojis to guide actions and shape perceptions.

Social Norms

influence individuals by giving information about the behavior of other users, provoking herd behavior

Colored Bar Progress

visual nudge showing goal progress and motivating completion.

Messenger effects

influence individuals by messages the users receive (e.g., warning symbols or reminder messages) [5]

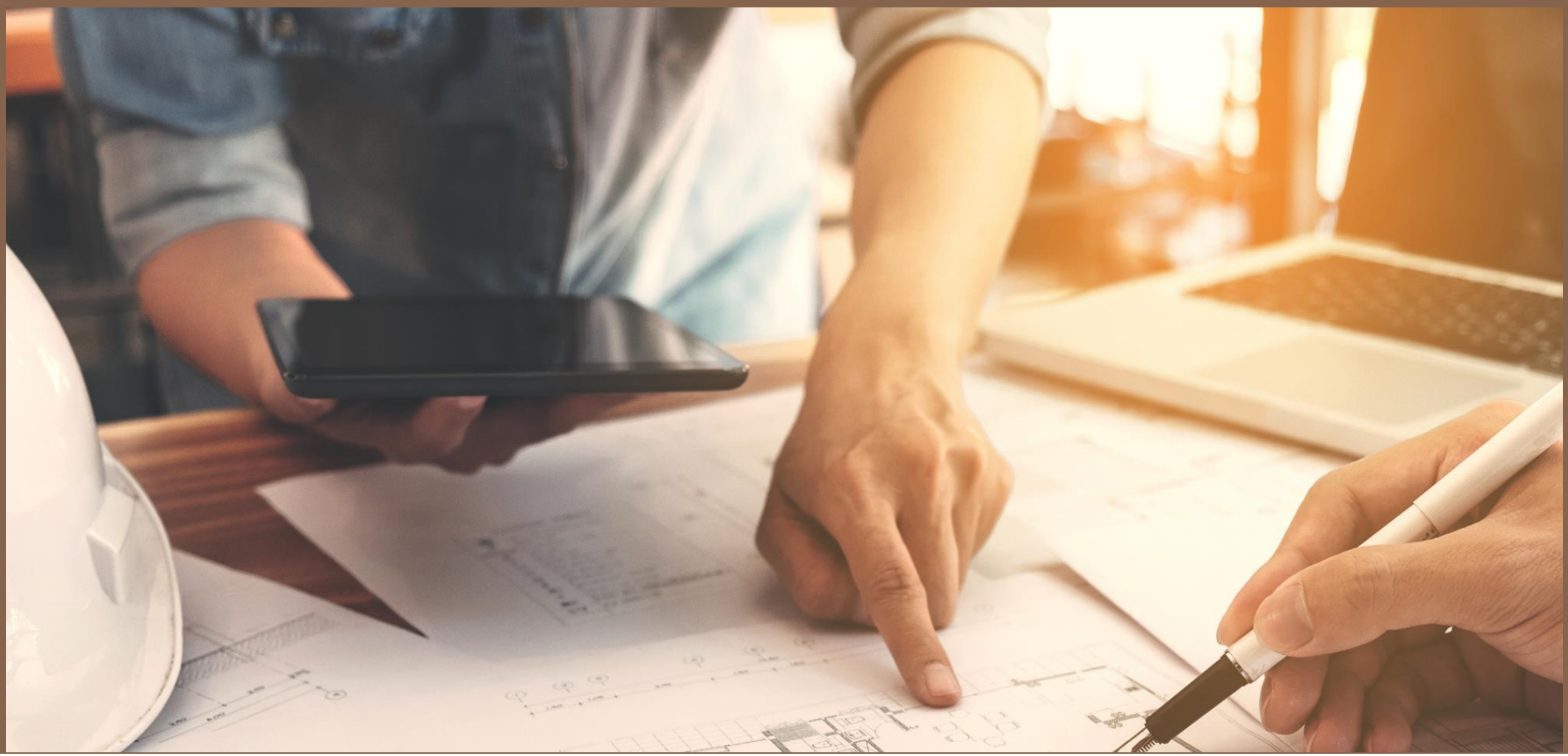

Conclusion

In conclusion, as aparticipants, we have the power to create positive change in our country through collaborative efforts.
By using our knowledge of science and technology to design digital nudges, we can motivate people towards action on issues such as social inclusion and gender equality.

The emotional connection we share as we work together towards a common goal is powerful, and the potential impact is nothing short of amazing.

Let us approach this work with creativity, passion, and a commitment to making our country a better place. And don't forget our Prophet Muhammad's saying, "Allah loves that when one of you does a job, he does it well."

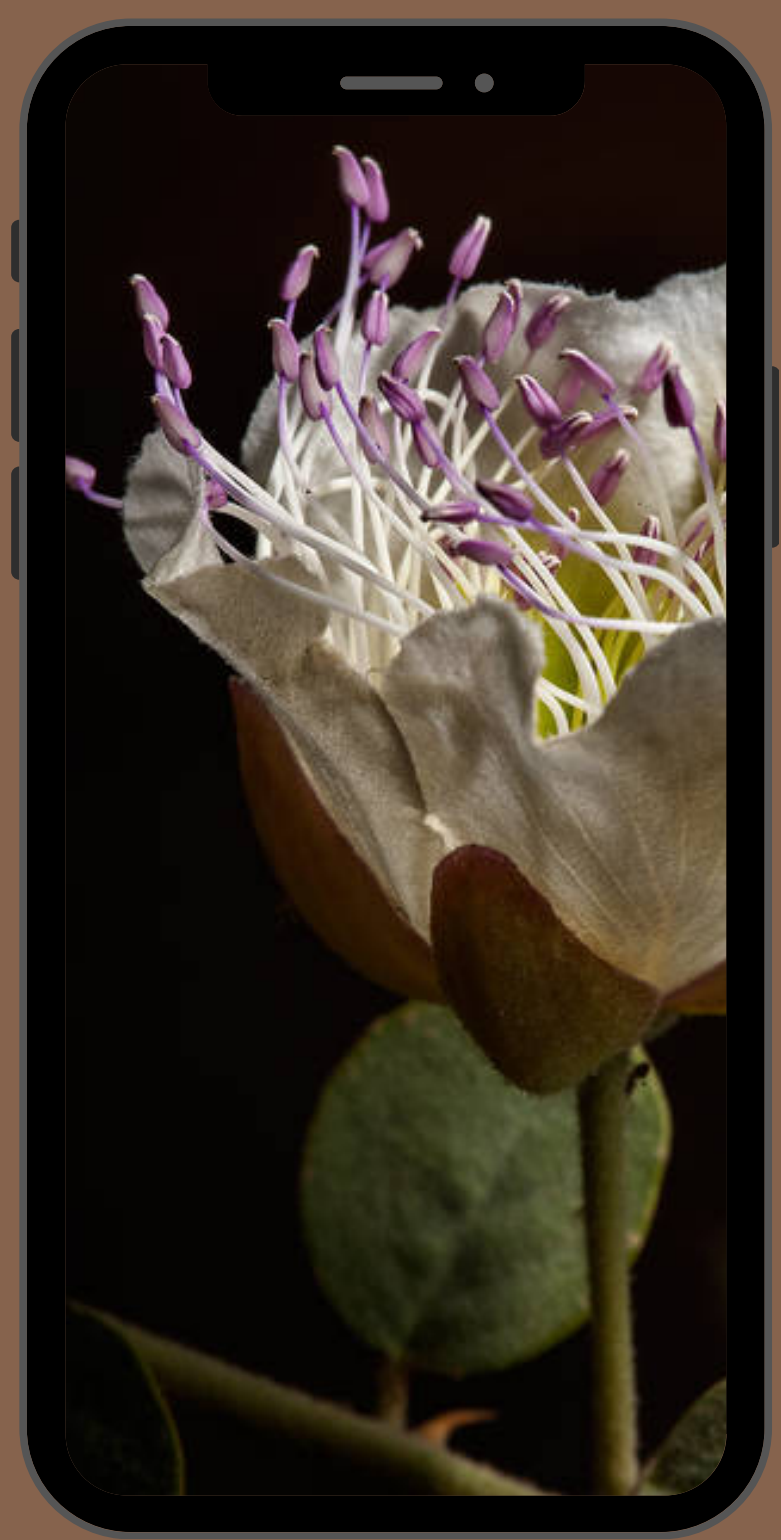

Refrences:

1-Sunstein, C. R. (2018). Nudging: a very short guide. In The Handbook of Privacy Studies (pp. 173-180). Amsterdam University Press

[2] Mirsch, T., Lehrer, C., & Jung, R. (2017). Digital nudging: Altering user behavior in digital environments. Proceedings der 13. Internationalen Tagung Wirtschaftsinformatik (WI 2017), 634-648

[3] Sunstein, C. R. (2018). Nudging: a very short guide. In The Handbook of Privacy Studies (pp. 173-180). Amsterdam University Press

[4] Valta, M., Menzel, J., Maier, C., Pflügner, K., Meier, M., & Weitzel, T. (2023). Digital Nudging: A Systematic Literature Review and Future Research Directions. In Proceedings of the 2022 Computers and People Research Conference. ACM. doi:10.1145/3510606.3550211.

[5]-Sunstein, C. R. (2018). Nudging: a very short guide. In The Handbook of Privacy Studies (pp. 173-180). Amsterdam University Press

Thank You

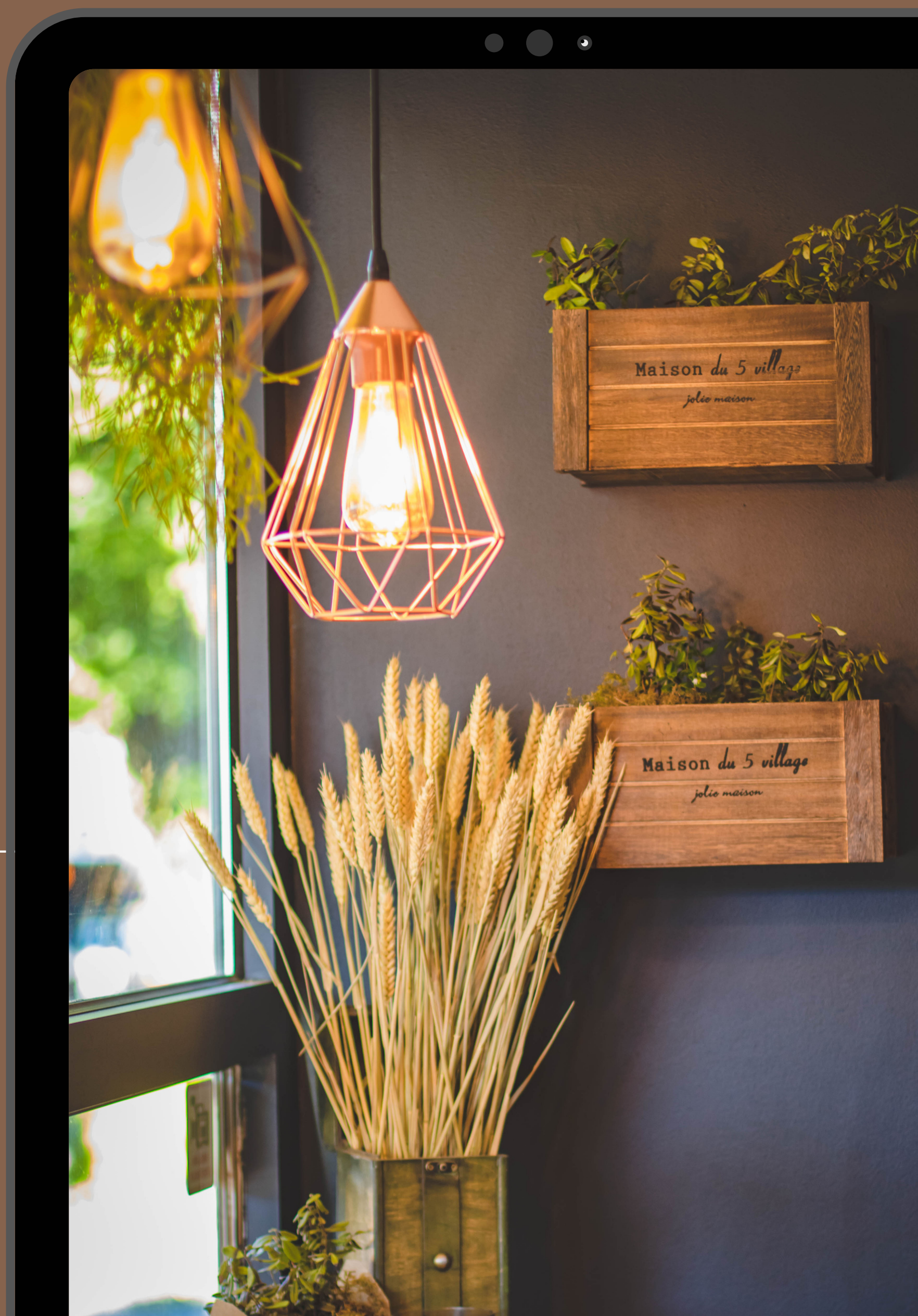

Supplement: Supplementary file 1 [file Data_Sheet_1.pdf]
