# Supplementary material for: Co-designing digital nudges to promote gender equality and social inclusion among Arab youth: a mixed-methods study
Source: Front Psychol. 2026 Sep 8;17:1763151. doi: 10.3389/fpsyg.2026.1763151 (PMC13593436; doi:10.3389/fpsyg.2026.1763151)
Supplement: Supplementary file 2 [file Data_Sheet_2.pdf]

SP#5 - GESI by Design

*Task 5.1.2: Conducting the co-design*

# Pilot Study Timeline

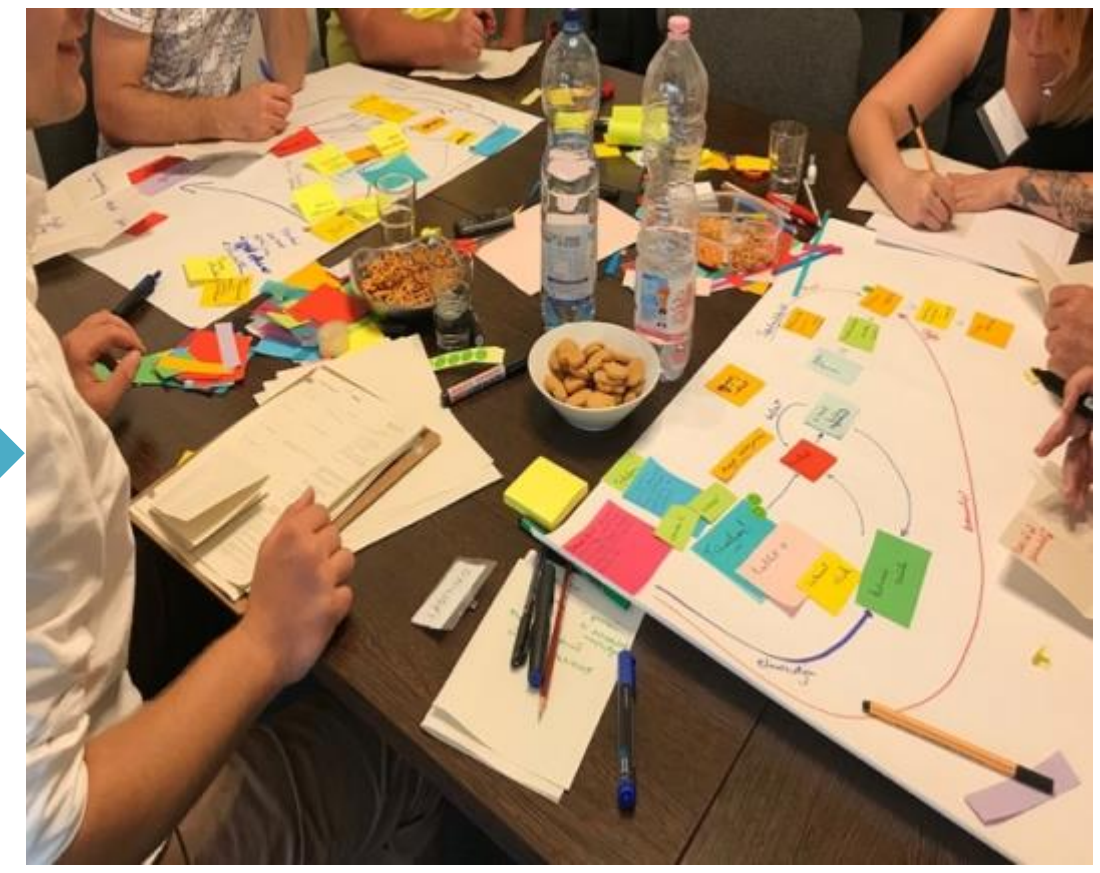

a detailed timeline for the pilot study, including start and end times. We will stick to the schedule as closely as possible.

## Main Phases

Defining
Workshop Goal

Designing
Digital Nudge

Gathering
Feedback

**Proposed Time: (9-11:00) am.**

Welcome → Problem Identification → Workshop Presentation → Booklet → Figma Examples → Design Based on Examples → Feedback and Discussion

| | Complete by | Each word in BOLD represents a file in the Study Design folder |
|---|---|---|
| ☐ | 9:20 am | Welcome and introduce myself, supervisors, and my research project to the participants, and thank them for attending |
| ☐ | 9:35 am | Identify the problem that the co-design session aims to address. |
| ☐ | 10:00 am | Present a **presentation** to ensure that participants gain a comprehensive understanding of key concepts, including Nudge, Digital Nudge, and GESI. |
| ☐ | 10:00 am | Provide participants with a **booklet** that contains essential information and guidelines relevant to the co-design activity |
| ☐ | 10:10am | Showcase **prototypes using Figma** to illustrate potential designs and walk participants through the examples to inspire their creativity. |
| ☐ | 10:30 | In groups, participants will go through a collaborative design exercise promoting GESI based on Figma examples |
| ☐ | 10:55 | Each group presents their designs in 4 minutes, followed by a vote for the top three, with winners receiving gifts |
| ☐ | 11:00 | Closure and Thanking the Participants |
